# Molecular and cellular basis of acid taste sensation in *Drosophila*

**DOI:** 10.1038/s41467-021-23490-5

**Published:** 2021-06-17

**Authors:** Tingwei Mi, John O. Mack, Christopher M. Lee, Yali V. Zhang

**Affiliations:** 1 Monell Chemical Senses Center, Philadelphia, PA USA; 2 Department of Biology, University of Pennsylvania, Philadelphia, PA USA; 3 Department of Physiology, The Diabetes Research Center, University of Pennsylvania, Perelman School of Medicine, Philadelphia, PA USA

**Keywords:** Sensory processing, Peripheral nervous system

## Abstract

Acid taste, evoked mainly by protons (H^+^), is a core taste modality for many organisms. The hedonic valence of acid taste is bidirectional: animals prefer slightly but avoid highly acidic foods. However, how animals discriminate low from high acidity remains poorly understood. To explore the taste perception of acid, we use the fruit fly as a model organism. We find that flies employ two competing taste sensory pathways to detect low and high acidity, and the relative degree of activation of each determines either attractive or aversive responses. Moreover, we establish one member of the fly Otopetrin family, Otopetrin-like a (OtopLa), as a proton channel dedicated to the gustatory detection of acid. OtopLa defines a unique subset of gustatory receptor neurons and is selectively required for attractive rather than aversive taste responses. Loss of *otopla* causes flies to reject normally attractive low-acid foods. Therefore, the identification of OtopLa as a low-acid sensor firmly supports our competition model of acid taste sensation. Altogether, we have discovered a binary acid-sensing mechanism that may be evolutionarily conserved between insects and mammals.

## Abstract (web summary)

Many animals, including mammals and insects, like slightly acidic yet dislike highly acidic foods, but how animals discriminate low from high acidity is unclear. Here the authors demonstrate that the fruit fly uses an evolutionarily conserved taste receptor to distinguish low from high concentrations of acid.

## Introduction

Sour taste, like sweet, bitter, salty, and umami tastes, represents a fundamental taste modality across many species ranging from insects to mammals^1,2^. Typically, we like slightly acidic foods such as lemon juice, which potentially indicates the presence of nutrients^3^. In contrast, we dislike highly acidic foods^4^, which can cause digestive tract tissue injuries^5^. The bivalent taste response to acid is also documented in rodents^6^. Similar to mammals, the fruit fly, *Drosophila melanogaster*, prefers low levels of acid, which stimulate feeding and reproduction^7,8^, and avoids high acid concentrations^9,10^. Therefore, although flies and humans appear drastically different, the hedonic valence of their acid-taste response is similar: it can be either attractive or aversive, depending on the acid concentration of food. We propose that the bidirectional characteristic of acid perception constitutes an evolutionary fitness that enables animals to choose nutritious and reject unhealthy food sources. How do animals make this seemingly challenging decision? We hypothesize that a taste-coding mechanism underlies the opposing feeding behavior triggered by low and high levels of acids. However, the molecular and cellular nature of the acid-taste coding has remained unclear.

Several lines of research demonstrate that type III taste receptor cells (TRCs) are responsible for acid sensing in mice^1,11^. Nevertheless, the type III TRC population may be heterogeneous and contain different cell subtypes. Due to the lack of molecular markers and genetic tools to manipulate different subsets of type III TRCs, the question of how type III TRCs differentially respond to different concentrations of acid appears to be difficult to address in mammals. Moreover, other than eliciting taste sensation, acid also activates trigeminal nerves in the oral cavity of mammals, leading to burning or pain sensation^12^. This side effect further confounds the investigation of sour-taste coding and sour-taste-triggered behavior in mammals. In contrast, our work has demonstrated that flies exhibit much more pronounced and distinct taste responses to varying concentrations of acid than do mammals. Therefore, the fly serves as an excellent animal model to elucidate the taste coding of acid. Here we report that the fly mainly uses two different subsets of gustatory receptor neurons (GRNs) to selectively sense low or high concentrations of acids. The taste transduction pathways orchestrated by low- and high-acid GRNs antagonize each other, and the net behavioral response to a particular concentration of acid is predominantly determined by the relative activities of low- vs. high-acid GRNs.

Animals take advantage of highly diversified taste receptors and TRCs to detect varying taste substances, including sugar, salt, acid, and bitter compounds^2,13^. In mammals, in contrast to the well-characterized sweet and bitter receptors, the molecular identity of sour-taste receptor had not been determined until a recent discovery showing that the Otopetrin (Otop) protein family functions as proton channels^14^. In mice, one of the Otop family members, Otop1, is essential for sour-taste transduction^15,16^. Despite this significant finding, the exact role played by Otop1 in discriminating low- from high-acid foods remained unclear. As the Otop family is fairly conserved between mammals and insects, we were curious if the Otop family is also required for taste sensation of acids in *Drosophila*, given that no bona fide sour-taste receptors had been established in insects. We discovered that one of the fly Otop orthologues, Otopetrin-like a (OtopLa), acts as a proton channel and is selectively required for attractive taste sensation of acids in *Drosophila*. The fly OtopLa protein is localized at the tip of the GRN dendrite, the forefront site of taste sensory cells that is responsible for directly sensing tastant stimuli. Further, OtopLa defines a novel class of GRNs, which are largely distinct from other groups of GRNs responding to sugar, salt, or bitter tastants. Furthermore, our genetic analysis showed that loss of *otopetrin-like a* (*otopla*) selectively abolishes the attractive acid-taste pathway, leaving the aversive pathway intact. Notably, the *otopla* mutant flies became abnormally averse to low concentrations of acid. Thus, we provide strong genetic evidence to establish not only that the attractive and aversive taste pathways responsible for acid sensation exist but also that they are genetically segregated. Finally, by establishing OtopLa as a bona fide taste receptor for acid in flies, our work overturns the long-standing view that insects and mammals use fundamentally different gustatory receptors.

## Results

**Fig. 1:**
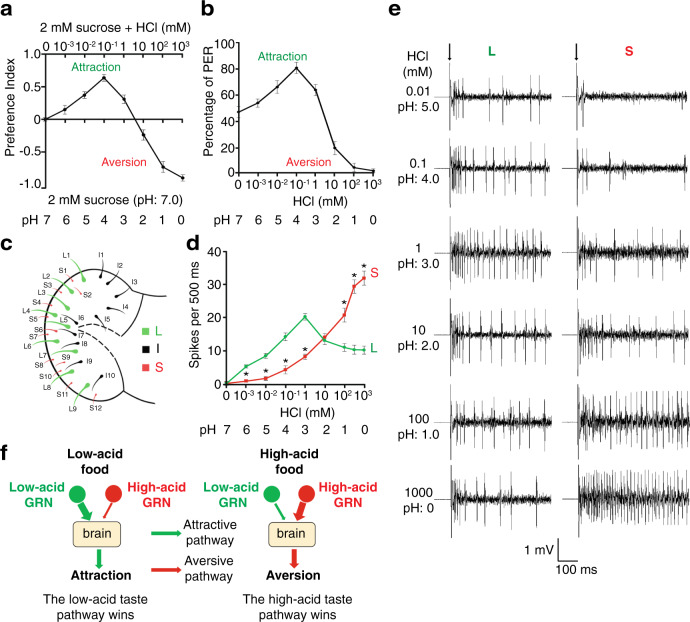
Gustatory responses to varying acid concentrations in distinct types of taste sensilla. **a** Feeding responses when choosing between 2 mM sucrose (pH 7.0) and 2 mM sucrose plus varying HCl concentrations (0.001–1000 mM). *n* = 12 trials, with about 70 animals tested in each trial. Data are presented as mean ± SEM. **b** Proboscis extension reflex (PER) in response to 30 mM sucrose food mixed with varying HCl concentrations. *n* = 12 trials (pH 3–6), *n* = 11 trials (pH 0–2), and *n* = 10 trials (pH 7). In each trial, at least ten animals were tested. Data are presented as mean ± SEM. **c** Distribution of taste sensilla on the proboscis, showing large (L), small (S), and intermediate (I) types. **d** Firing rates of L6 and S5 sensilla in response to a series of HCl concentrations. Data are presented as mean ± SEM. *n* = 10 animals, *p < 0.0001 (0.001, 0.01, 0.1, 1, and 1000 mM) and *p = 0.0006 (100 mM), unpaired two-tailed *t*-tests. **e** Representative action potentials evoked by a broad range of HCl concentrations at L6 and S5 sensilla. Arrows indicate the onset of taste stimuli. **f** Competition model of acid-taste coding.

We employed the fly as a model organism to decipher the taste sensation of acid, given that it has been used successfully over the past 20 years to identify taste receptors, sensory cells, and neuronal circuits involved in taste perception^17–19^. To quantitatively study the fly’s feeding responses to different concentrations of strong acids, such as hydrochloric acid (HCl), we conducted the two-way feeding assay, a well-established assay in *Drosophila*^20–23^. In brief, we allowed wild-type flies to choose between neutral foods and acidic foods containing various concentrations of HCl (0.001–1000 mM). Based on our feeding assays, wild-type flies prefer slightly acidic foods and reject highly acidic food options (Fig. 1a). To directly measure the wild-type fly’s taste response to foods containing different concentrations of HCl, we employed the proboscis extension reflex (PER) assay^21^. In this assay, we applied a drop of acidic solution to the fly’s proboscis, a primary taste organ analogous to the tongue in mammals, and immediately monitored the extension response of the proboscis. Consistent with the results obtained through the two-way feeding assay, we found that the wild-type fly exhibits strong PER responses when low concentrations of HCl are applied to the proboscis, indicating an attractive feeding response. In contrast, the wild-type fly shows few PER responses when stimulated with higher concentrations of acids (Fig. 1b). Along with previous work^10^, our behavioral assays demonstrate that wild-type flies display a bidirectional response to foods with varying concentrations of acids: attractive to low-acid foods but aversive to high-acid foods.

We hypothesized that there are two different groups of GRNs in the proboscis, which account for the opposite behavioral responses to low- and high-acid-containing foods. To test this idea, we performed tip recordings, a highly robust electrophysiological technique for interrogating the physiology of GRNs^20,22,23^. Thirty-one taste sensilla decorate the surface of the fly labellum. Based on taste function, location, and morphology, these sensilla are classified into three types: 9 large (L), 12 small (S), and 10 intermediate (I) sensilla (Fig. 1c)^24,25^.

First, we were curious about the spatial coding of acid taste—how do different types of sensilla differentially respond to low or high concentrations of acid? To address this question, we conducted a comprehensive survey of the 31 sensilla with low acid (1 mM HCl) and high acid (100 mM HCl) as the taste stimuli. As revealed by our tip recordings, among the three groups of sensilla, the L-type sensilla, such as L6, fire high-frequency action potentials in response to 1 mM HCl (Supplementary Fig. 1). In contrast, the S-type sensilla show weakest responses to 1 mM HCl. Conversely, the S-type sensilla, such as S5, elicit many more action potentials than the L-type when stimulated by 100 mM HCl (Supplementary Fig. 1). In addition, the I-type sensilla, when stimulated by low or high concentrations of acid, display weaker responses than the L- or S-type sensilla, respectively. Taken together, we conclude that the L-type sensilla predominantly respond to low concentrations of acid, whereas the S-type sensilla show the maximal response to high concentrations of acid.

Next, we explored the intensity coding of acid taste—how does each taste sensillum decode a broad range of acid concentrations? To solve this problem, we chose the L6 and S5 sensilla as representative examples to examine how L- and S-type sensilla respond to a series of HCl concentrations, ranging from very low (0.001 mM) to very high (1000 mM). According to our tip recordings, when we stimulated the sensilla with low concentrations of acid (0.001–1 mM), the number of action potentials fired by L6 sensilla rapidly increases and reaches a peak at around 1 mM (Fig. 1d, e). In contrast, S5 sensilla produce a significantly lower frequency of spikes than L6 under these conditions. This suggests that L6 sensilla are selectively tuned to low concentrations of acid. As the acid concentration rises, the spike frequency of L6 sensilla declines, implying that their acid-taste receptors have been saturated. Remarkably, when stimulated by high levels of acid (e.g., 100 mM), the S5 sensilla exhibit a considerable increase in the firing frequency, which becomes much higher than that of L6 sensilla. Thus, our results suggest that the S5 sensillum is optimally tuned to high concentrations of acid.

In light of these findings, we propose that there are two acid-sensing pathways mediated by attractive GRNs and aversive GRNs in the fly proboscis (Fig. 1f). Foods containing low concentrations of acid preferentially activate the L- over S-type sensilla, leading to animals perceiving the food as attractive. Conversely, when high concentrations of acids are encountered, robust stimulations of the S-type sensilla override the input from L-type sensilla, driving the fly’s rejection of high-acid foods.

As the bona fide acid-taste sensor has yet to be established in *Drosophila*, we turned our attention to the Otop protein family, which is evolutionarily conserved across diverse phylogenies. Recent work in mouse models reports that an Otop family member, Otop1, forms a proton-conducting ion channel and is required for taste sensation of acids^14,15^. Thus, the Otop family is an excellent candidate for sour-taste sensors in *Drosophila*. We used RNA interference (RNAi) to knock down expression of three fly *otop* genes, *otopla*, *otopetrin-like b* (*otoplb*), and *otopetrin-like c* (*otoplc*), specifically in GRNs using a *poxn-Gal4* line^26^. Knocking down *otopla*, but not *otoplb* or *otoplc*, leads to severe impairments in the preference for low-acid foods (Supplementary Fig. 2a) but has little effect on the aversive feeding responses to high concentrations of acid (Supplementary Fig. 2b).

**Fig. 2:**
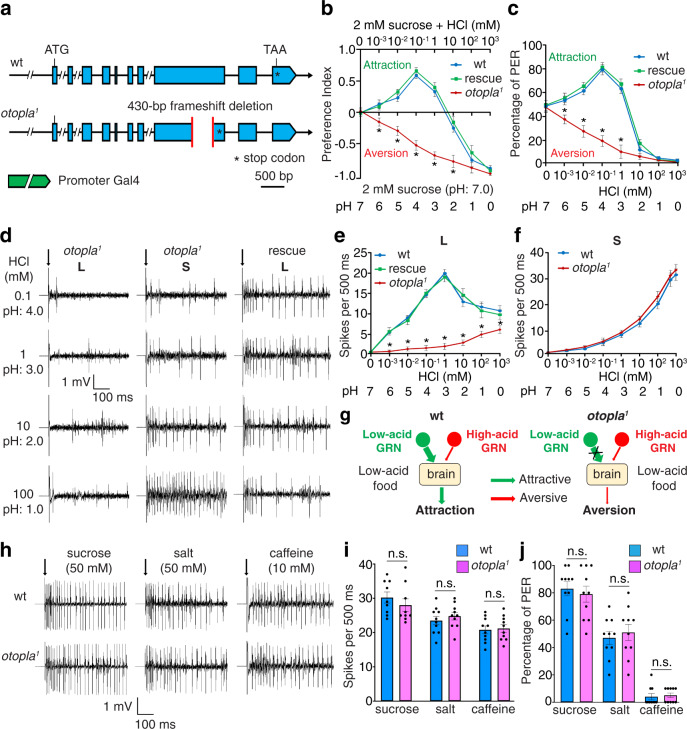
Gustatory responses to acid and other tastants in wild-type (wt), *otopla*^*1*^ mutant, and rescue (*otopla*^*1*^; *otop-Gal4/UAS-otopla*) flies. **a** Genomic structures of wild-type and mutant *otopla* genes. * Represents stop codon. **b** Feeding responses to neutral foods vs. foods with varying HCl concentrations. Data are presented as mean ± SEM. *n* = 12 trials, *p = 0.007 (0.001 mM) and *p < 0.0001 (0.01, 0.1, 1, and 10 mM), one-way ANOVA tests. **c** PER responses to 30 mM sucrose with varying concentrations of HCl. Data are presented as mean ± SEM. *n* = 12 trials, *p = 0.0039 (0.001 mM) and *p < 0.0001 (0.01, 0.1, and 1 mM), one-way ANOVA tests. **d** Representative tip recording traces of L6 and S5 sensilla. Arrows indicate the onset of taste stimuli. **e**, **f** Dose–response curves showing spikes elicited by different concentrations of HCl in L6 sensilla (**e**; *n* = 10 animals, *p < 0.0001 (0.001–100 mM) and *p = 0.0004 (1000 mM), one-way ANOVA tests) and S5 sensilla (**f**; *n* = 10 animals). Data are presented as mean ± SEM. **g** Model of aberrant acid-taste coding in *otopla*^*1*^. **h**, **i** Tip recordings responding to sucrose (50 mM; *n* = 9 animals), NaCl (50 mM; *n* = 10 animals), and caffeine (10 mM; *n* = 10 animals). Arrows in **h** indicate the onset of taste stimuli. Data in **i** are presented as mean ± SEM. n.s., not significant, unpaired two-tailed *t*-tests. **j** PER assays responding to sucrose (50 mM; *n* = 10 trials), NaCl (50 mM; *n* = 10 trials), and caffeine (10 mM; *n* = 10 trials). Data are presented as mean ± SEM. n.s., not significant, unpaired two-tailed *t*-tests.

To genetically determine if fly *otopla* is required for acid-taste sensation, we generated a null mutant for *otopla* using the CRISPR/Cas9 technique^27^. The fly OtopLa protein is predicted to have 12 transmembrane segments. Accordingly, we designed two guide RNAs (gRNAs) that target two loop regions flanking the sixth transmembrane (TM6) segment of the OtopLa protein. gRNA-targeted DNA cleavage can delete the TM6 segment, as well as parts of the flanking loop regions. We successfully obtained an *otopla* deletion mutant, *otopla*^*1*^, in which 430 base pairs (bp) of the protein-coding sequences were removed (Fig. 2a; see also Supplementary Fig. 3a–c). In addition, the 430 bp out-of-frame deletion results in frameshift mutations for the remaining OtopLa protein-coding sequences. Therefore, we concluded that *otopla*^*1*^ represents a loss-of-function mutant.

We found that the *otopla*^*1*^ mutant develops normally to the adult stage. In addition, the motor activity of *otopla*^*1*^ mutant appears indistinguishable from wild type. However, unlike wild-type flies, the *otopla*^*1*^ mutants show aversive responses to low acid, while still exhibiting normal avoidance of high acid (Fig. 2b). To determine whether aversion to low acid arises from a functional defect in the taste organ, we performed the PER assay. *Otopla*^*1*^ mutants exhibit considerably reduced PER responses to low (Fig. 2c) rather than high acidity compared to wild type, suggesting *otopla*^*1*^ mutants perceive otherwise attractive low acid as aversive. To examine whether the mutant phenotype is solely due to the loss of *otopla*, we made a promoter-*Gal4* line, *otopla-Gal4*, as well as a *UAS-otopla* line. Using the *UAS/Gal4* system^28^, we found that the mutant phenotype can be restored to wild type in *otopla*^*1*^; *otopla-Gal4/UAS-otopla* animals, where the wild-type *otopla* is expressed in the *otopla*^*1*^ mutant background under the control of *otopla*-*Gal4* (Fig. 2b, c).

Why does loss of *otopla* convert the taste quality of low acid from attractive to aversive? We reasoned that loss of *otopla* may unbalance the competition between the attractive and aversive acid-taste pathways. To test this, we conducted tip recordings for wild-type and *otopla*^*1*^ mutant flies. Initially, we stimulated the L6 sensilla with a series of HCl concentrations. The L6 sensilla of *otopla*^*1*^ mutants fire significantly fewer action potentials than wild type (Fig. 2d, e). However, there is no significant difference in the firing frequency of the S5 sensilla between wild-type and *otopla*^*1*^ flies in response to either high or low concentrations of acid (Fig. 2d, f). In accordance with our model, loss of *otopla* selectively inactivates the attractive pathway and leaves the aversive pathway unimpaired, causing mutant flies to reject both low- and high-acid foods (Fig. 2g). Thus, our genetic analysis of *otopla* firmly supports that attractive and aversive pathways for the acid-taste sensation not only exist but also are genetically segregated, with *otopla* being specifically required for attraction to mild acidity.

Moreover, we found that *otopla*^*1*^ mutants show normal electrophysiological responses to sweet (50 mM sucrose), salty (50 mM NaCl), and bitter (10 mM caffeine) tastants (Fig. 2h, i). Using the PER assays (Fig. 2j), we found that the *otopla*^*1*^ mutants exhibit normal attractive responses to sugar and low concentrations of salt and normal aversive responses to the bitter tastant caffeine. In summary, our results demonstrate that fly *otopla* is selectively required for the attractive taste response to low acid.

**Fig. 3:**
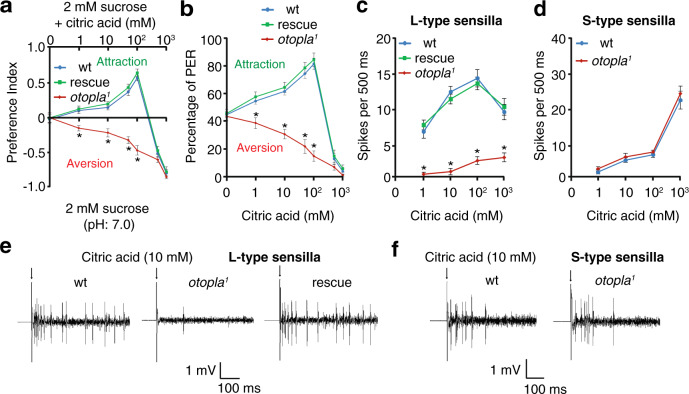
Feeding and electrophysiological responses to low and high concentrations of citric acid for wild-type (wt), *otopla*^*1*^ mutant, and rescue flies. **a** Feeding preference between neutral foods (2 mM sucrose) and foods comprising 2 mM sucrose and varying concentrations of citric acid (1 mM, pH 3.7; 10 mM, pH 3.3; 100 mM, pH 2.8; 1000 mM, pH 2.0). Data are presented as mean ± SEM. *n* = 10 trials, *p = 0.0001 (1 mM) and *p < 0.0001 (10, 50, and 100 mM), one-way ANOVA tests. **b** Proboscis extension reflex (PER) responses to foods containing 30 mM sucrose and a series of concentrations of citric acid. Data are presented as mean ± SEM. *n* = 10 trials, *p = 0.0007 (1 mM) and *p < 0.0001 (10, 50, and 100 mM), one-way ANOVA tests. **c**, **d** Statistical analyses of spike frequencies evoked by different concentrations of citric acid at the L6 sensilla (**c**; *n* = 10 animals, *p < 0.0001 (1–1000 mM), one-way ANOVA tests) and S5 sensilla (**d**; *n* = 10 animals). Data are presented as mean ± SEM. **e**, **f** Representative spike trains elicited by 10 mM citric acid at the L6 sensilla (**e**) and S5 sensilla (**f**). Arrows indicate the onset of taste stimuli.

Next, we wanted to know whether the acid-taste coding mechanism uncovered using the strong acid HCl also applied to weak acids. To rule out the interference of acid sensing from the olfactory system, we chose citric acid, a naturally occurring nonvolatile weak organic acid enriched in citrus fruits such as lemons and oranges^29^. We conducted two-way feeding and PER assays using varying concentrations of citric acid (1–1000 mM). Wild-type flies strongly prefer low levels of citric acid (e.g., 10 mM) but significantly avoid high concentrations (1000 mM). Similar to the response to HCl, *otopla*^*1*^ mutant flies are averse to low concentrations of citric acid, suggesting that loss of *otopla* selectively disrupts the attractive pathway for citric acid (Fig. 3a, b). Furthermore, we performed tip recording analyses and found that, compared to wild type, *otopla* mutant flies exhibited a significant reduction in the number of action potentials evoked by varying concentrations of citric acid at the L6 sensilla, whereas no significant changes were detected in the S5 sensilla (Fig. 3c–f).

**Fig. 4:**
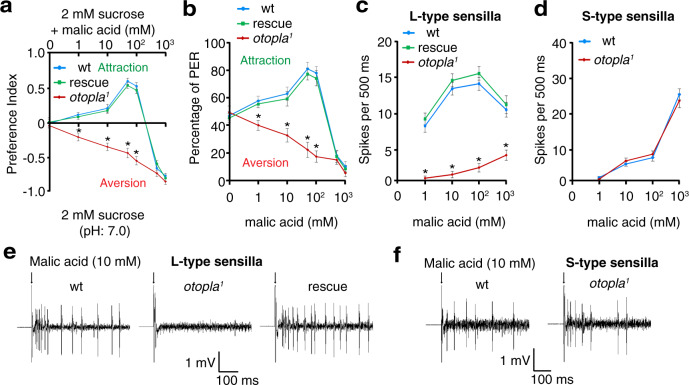
Feeding and electrophysiological responses to low and high concentrations of malic acid among wild-type (wt), *otopla*^*1*^ mutant, and rescue flies. **a** Feeding preference between neutral foods containing 2 mM sucrose and foods comprising 2 mM sucrose and malic acid (1 mM, pH 3.6; 10 mM, pH 3.2; 100 mM, pH 2.7; 1000 mM, pH 1.9). Data are presented as mean ± SEM. *n* = 10 trials, *p < 0.0001 (1–100 mM), one-way ANOVA tests. **b** PER responses to foods containing 30 mM sucrose and different concentrations of malic acid. Data are presented as mean ± SEM. *n* = 10 trials, *p = 0.0012 (1 mM), *p = 0.0004 (10 mM), and *p < 0.0001 (50 and 100 mM), one-way ANOVA tests. **c**, **d** Statistical analyses of spike frequencies evoked by varying concentrations of malic acid at the L6 sensilla (**c**; *n* = 10 animals (1–1000 mM), *p < 0.0001 (1–1000 mM), one-way ANOVA tests) and S5 sensilla (**d**; *n* = 10 animals for 1 and 10 mM, *n* = 8 animals for 100 mM, and *n* = 9 animals for 1000 mM). Data are presented as mean ± SEM. **e**, **f** Representative spike trains elicited by 10 mM malic acid at the L6 sensilla (**e**) and S5 sensilla (**f**). Arrows indicate the onset of taste stimuli.

To further examine the generality of our acid-taste coding model, we tested another nonvolatile organic acid, malic acid, which is also found mainly in fruits, such as apples^30^. Similarly, wild-type flies liked low concentrations of malic acid but disliked high concentrations (Fig. 4a, b). However, *otopla*^*1*^ mutant flies show abnormally aversive responses to low concentrations of malic acid (Fig. 4a, b). Moreover, our tip recordings revealed that loss of *otopla* selectively affects the firing of L6 sensilla in response to malic acid, while leaving the S5 sensilla intact (Fig. 4c–f). Collectively, our behavioral and physiological analyses of taste sensations evoked by HCl, citric acid, and malic acid indicate that flies employ a similar taste-coding strategy to discriminate low from high concentrations of both strong and weak acids.

**Fig. 5:**
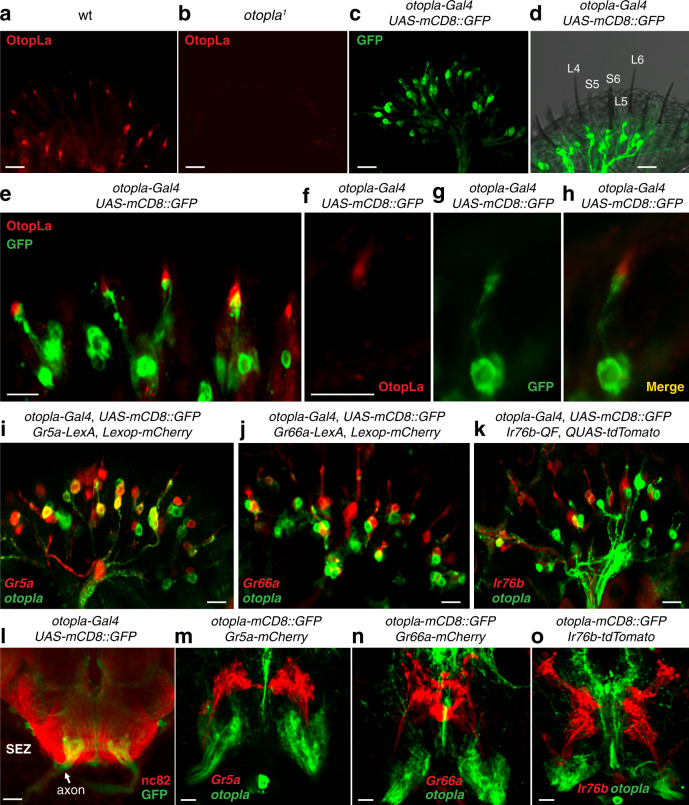
Expression patterns of *otopla* in the fly taste system. **a**, **b** Immunoreactivity of anti-OtopLa in wild-type (wt; **a**) and *otopla*^*1*^ (**b**) proboscises. **c** Expression of *otopla-Gal4* in the labellum. **d** Distribution of L- and S-type taste sensilla relative to the dendrites of *otopla-*expressing GRNs in the proboscis. **e** Double labeling of anti-OtopLa and anti-GFP in *otopla-Gal4*; *UAS-mCD8::GFP* flies. **f**–**h** Localization pattern of OtopLa in a single GRN dendrite. **i**–**k** Localization of *otopla*-positive sour GRNs relative to *Gr5a*-positive sweet GRNs (**i**), *Gr66a*-positive bitter GRNs (**j**), and *Ir76b*-positive salty GRNs (**k**). **l** Projection pattern of *otopla*-positive GRNs in the subesophageal zone (SEZ). Arrow indicates *otopla*-positive GRN axon bundles. **m**–**o** SEZ projections of *otopla*-positive GRNs relative to *Gr5a*-positive GRNs (**m**), *Gr66a*-positive GRNs (**n**), and *Ir76b*-positive GRNs (**o**). All immunocytochemical experiments were repeated at least three times with similar results. Scale bars: 20 µm (**a**–**d**, **m**–**o**), 10 µm (**e**–**k**), 50 µm (**l**).

Another key question concerning the role of OtopLa in sour-taste transduction is its expression pattern. In *Drosophila*, the GRN, a bipolar type of neuron, is the primary TRC^2^. The GRN extends a short dendrite into the luminal space of the sensillum to directly sense the tastants dissolved from the food environment^31^. To examine the endogenous expression pattern of OtopLa, we made polyclonal antibodies against a peptide located between the transmembrane segment 5 (TM5) and TM6 regions of OtopLa protein. We performed immunocytochemistry with OtopLa antibodies and imaged the  samples with confocal microscopy. Of great interest, we found that OtopLa is expressed in a subset of GRNs in the proboscis (Fig. 5a). In contrast, little immunoreactivity was detected in *otopla*^*1*^ mutant flies (Fig. 5b), indicating the specificity of the antibody. To gain deeper insight into the expression patterns of *otopla*, we created a promoter-*Gal4* transgenic line, *otopla-Gal4*. After crossing the *otopla-Gal4* line to a membrane-tethered green fluorescent protein (GFP) reporter line such as the *UAS-mCD8::GFP*^32^, we examined the *otopla-Gal4* expression pattern. The *otopla-Gal4* expression was readily detected in a subset of GRNs in the fly proboscis (Fig. 5c). Notably, the *otopla*-expressing GRNs mostly innervate L- rather than S-type sensilla (Fig. 5d), providing compelling evidence to support that low acid is mainly detected by L-type sensilla. After performing the double-labeling analysis of the *otopla-Gal4/UAS-mCD8::GFP* fly, we found that OtopLa is enriched in the tip of the GRN dendrite, the forefront site responsible for detecting taste compounds from the food environment (Fig. 5e–h). Therefore, our cell biological data indicate that OtopLa is a direct acid-taste sensor. Moreover, our results demonstrate that the expression patterns of endogenous *otopla* and *otopla*-*Gal4* overlap (Fig. 5e–h), suggesting that expression of *otopla*-*Gal4* faithfully recapitulates that of endogenous *otopla*.

We also determined the relative expression pattern between acid-sensing GRNs and sweet, bitter, or salty GRNs. Toward this goal, we used the *Gal4/UAS* system to label the *otopla*-expressing GRNs and the *LexA/LexOP*^28,33^ system to highlight sweet or bitter GRNs that are defined by *Gr5a-LexA* or *Gr66a-LexA*, respectively^34,35^. Specifically, we examined the expression of *otopla*-positive GRNs relative to sweet GRNs labeled by *Gr5a-LexA* (Fig. 5i) or bitter GRNs labeled by *Gr66a-LexA* (Fig. 5j). In addition, we chose the *Q* system^36^ to label salty GRNs, which are marked by *Ir76b-QF*^22^ and *QUAS-tdTomato* (Fig. 5k). Our double-labeling and confocal microscopy analyses demonstrated that the *otopla*-expressing GRNs are mostly separate from sweet, bitter, and salty GRNs in the proboscis, although a small fraction (around 15%) of *otopla*-expressing GRNs appear to overlap with sweet GRNs. Thus, our results suggest that the *otopla*-expressing GRNs essentially represent a distinct group of GRNs. Finally, we examined the central projection patterns of *otopla-*expressing GRNs in the subesophageal zone (SEZ), a fly brain region receiving taste input from the peripheral taste organ (Fig. 5l). We found that *otopla*-expressing GRNs mainly project to a unique area situated in the ventral lateral region of the SEZ. Consistent with the localization pattern at the periphery, the *otopla*-expressing GRN-projecting region in the SEZ segregates from the regions innervated by sweet, bitter, and salty GRNs (Fig. 5m–o). In conclusion, we discovered that *otopla* is expressed in a previously unidentified group of GRNs.

**Fig. 6:**
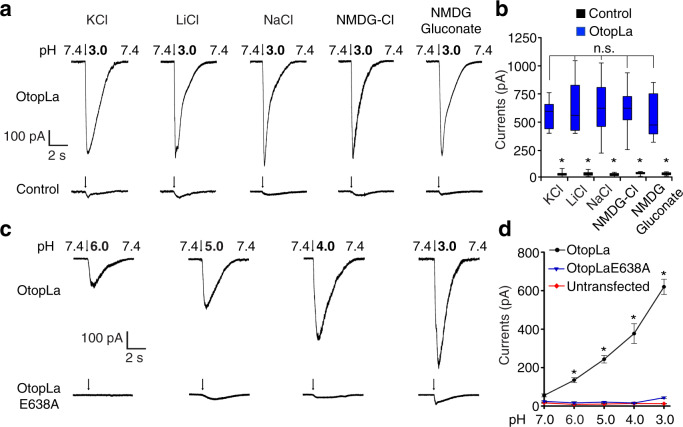
Currents evoked by acidic pH in HEK293 cells expressing OtopLa or OtopLaE638A. **a** Representative currents produced by the stimuli of acidic isosmotic solutions (pH 3.0) of KCl, LiCl, NaCl, NMDG-Cl, or NMDG-gluconate in HEK293 cells with or without OtopLa expression. Arrows indicate the onset of stimuli. **b** Statistical analysis of current amplitudes in HEK293 cells with or without OtopLa expression in various bath solutions containing KCl, LiCl, NaCl, NMDG-Cl, or NMDG-gluconate. *n* = 11 cells, *p < 0.0001 for all bath solutions, unpaired two-tailed *t*-tests. One-way ANOVA tests comparing current amplitudes of OtopLa-expressing cells in KCl, LiCl, NaCl, NMDG-Cl, and NMDG-gluconate bath solutions gave *p* = 0.95, indicating no significant differences among these five bath solutions. In the box and whisker plot, top and bottom whiskers represent the maximum and the minimum, respectively; top and bottom box boundaries represent third and first quartiles of the data, respectively; and the center line represents the median. **c**, **d** Currents elicited by acidic isosmotic solutions (pH 3.0–6.0) in HEK293 cells expressing OtopLa or OtopLaE638A, and controls. NMDG-gluconate as bath solution. Arrows in **c** indicate the onset of stimuli. Data are presented as mean ± SEM. *n* = 12 cells, *p < 0.0001 (pH 3.0–6.0), one-way ANOVA tests.

Our behavioral, anatomical, and physiological results strongly suggest that OtopLa is necessary for acid-taste sensation. Another critical question we wanted to address is whether OtopLa is sufficient to be a bona fide taste receptor for acid. Despite previous work showing that fly Otop-like c (OtopLc) can form proton channels in heterologous cells^14^, it remained unclear whether OtopLa, which has a low level of protein sequence homology (about 30%) with OtopLc, is sufficient to form proton channels. To examine whether the OtopLa protein can be activated by protons in vitro, we performed the patch-clamp analysis of OtopLa expressed in the HEK293 heterologous cell line. To determine whether OtopLa proteins can localize at the HEK293 cell surface, we fused a Myc tag to the N terminus of the OtopLa protein. Using antibodies against Myc and our antibodies against a loop region between the TM5 and TM6 segments of OtopLa protein (Supplementary Fig. 4a), we performed immunocytochemical assays when the HEK293 cells were permeabilized with Triton X-100 detergent and when not permeabilized without detergent. Our immunocytochemical analyses showed that OtopLa proteins successfully traffic to the cell surface, as detected by OtopLa antibodies with or without detergent (Supplementary Fig. 4b–f), making the whole-cell patch-clamp recording feasible. Moreover, no obvious immunostaining signals were detected by anti-Myc antibodies when the cells were not permeabilized, suggesting that the N terminus of OtopLa is located intracellularly, while the loop between TM5 and TM6 is outside the cell. Under physiological conditions (pH 7.4), we failed to observe any obvious currents in cells expressing OtopLa. Notably, when we stimulated OtopLa-expressing cells with isotonic acidic solutions (pH 3.0), a considerably robust current was detected, whereas little current was detected in control cells without OtopLa (Fig. 6a, b). Thus, our patch-clamp recordings suggest that OtopLa forms an ion channel that can be activated by acidic pH in vitro.

To determine the ion selectivity of the OtopLa ion channel, we tested a series of bath solutions containing KCl, LiCl, NaCl, *N*-methyl-d-glucamine (NMDG)-Cl (the large cation NMDG does not pass through the plasma membrane), and NMDG-gluconate, which is completely membrane impermeable due to the addition of the large anion gluconate. Remarkably, even with the bath solution composed of membrane-impermeable NMDG-gluconate, we still detected a strong inward current evoked by mildly acidic pH (pH 3.0). All ions elicited comparable levels of inward currents in isotonic acidic bath solutions (pH 3.0) (Fig. 6a, b), indicating that the OtopLa channel is mainly conductive to protons. The amplitude of inward currents generated by the wild-type OtopLa channel significantly increases as the proton concentration in the bath rises (pH 6.0–3.0) (Fig. 6c, d). To conduct structural and functional analyses of OtopLa channels, we inspected all amino acid residues within 12 transmembrane segments and found only one negatively charged glutamate residue, E638 embedded in TM6. Furthermore, the E638 residue of OtopLa is evolutionarily conserved among worms, flies, mice, and humans (Supplementary Fig. 4a). Thus, we reasoned that this negatively charged E638 is likely involved in proton conduction through electrostatic interactions. To examine the role of E638, we mutated it to a nonpolar alanine residue. We performed immunostaining in the presence and absence of Triton X-100 detergent and found that OtopLaE638A proteins are also able to traffic to the cell surface of HEK293 cells (Supplementary Fig. 4g–k). However, the inward currents evoked by acidic solutions in OtopLaE638A-expressing cells were barely detectable compared to cells expressing wild-type OtopLa (Fig. 6c, d). In conclusion, the E638 residue of the OtopLa channel is key to proton conductance.

**Fig. 7:**
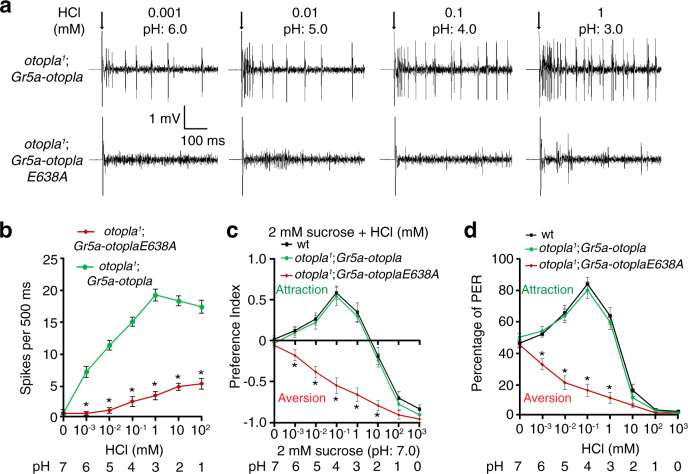
Responses to acid in flies misexpressing OtopLa or OtopLaE638A in sweet GRNs. **a**, **b** Action potentials evoked by various concentrations of HCl at L8 sensilla in flies misexpressing OtopLa or OtopLaE638A in *Gr5a*-positive GRNs. Arrows indicate the onset of taste stimuli. Data are presented as mean ± SEM. *n* = 10 animals, *p < 0.0001 (0.001–100 mM), unpaired two-tailed *t*-tests. **c** Feeding responses to neutral foods vs. foods with varying concentrations of HCl for wild-type flies and flies misexpressing OtopLa or OtopLaE638A. Data are presented as mean ± SEM. *n* = 10 trials, *p = 0.0029 (0.001 mM) and *p < 0.0001 (0.01, 0.1, 1, and 10 mM), one-way ANOVA tests. **d** PER responses to neutral and acidic foods in wild-type flies and flies misexpressing OtopLa or OtopLaE638A. Data are presented as mean ± SEM. *n* = 10 trials, *p < 0.0001 (0.001, 0.01, 0.1, and 1 mM), one-way ANOVA tests.

Finally, to establish OtopLa as an acid-taste receptor in vivo, we tested whether the expression of OtopLa in normally acid-blind sweet GRNs labeled by *Gr5a-Gal4*^37^ is sufficient to confer acid sensitivities to these GRNs. To eliminate the interference of endogenous OtopLa, experiments were done in an *otopla*^*1*^ background. We examined the localization of wild-type OtopLa and OtopLaE638A mutant expressed in sweet GRNs and found that both wild-type and mutant versions are able to traffic to the dendrite tip of the sweet GRN (Supplementary Fig. 5a, b). To determine whether protons can induce action potentials in sweet GRNs that express OtopLa, we conducted tip recordings on the L8 sensilla, which respond weakly to acids in wild-type animals (Supplementary Fig. 1). Interestingly, the OtopLa-misexpressing sweet GRNs yield robust action potentials when stimulated by low concentrations of HCl. In contrast, sweet GRNs misexpressing OtopLaE638A yield few action potentials (Fig. 7a, b). As controls, sweet GRNs misexpressing OtopLa and OtopLaE638A fired similar levels of sucrose-evoked action potentials (Supplementary Fig. 6), suggesting that the failure to respond to acid in OtopLaE638A-misexpressing flies is not due to nonspecific dysfunctions of sweet GRNs. Finally, flies misexpressing wild-type OtopLa, but not OtopLaE638A, are attracted to mildly acidic foods in the two-way feeding assays (Fig. 7c) and PER assays (Fig. 7d).

## Discussion

We have discovered an acid-taste coding mechanism that accounts for opposing taste responses to low and high acidity in a wide variety of animals, including mammals. Moreover, we have established that fly OtopLa, akin to its mammalian ortholog Otop1, is both necessary and sufficient for the attractive acid-taste sensation in *Drosophila*. To our knowledge, fly OtopLa is the first taste receptor identified in insects that has a direct ortholog in mammals. As the taste receptors dedicated to acid sensation between flies and mammals are well conserved, our fly work provides a practical avenue for fully understanding the acid-taste sensation in mammals, including humans.

According to our behavioral assays, the wild-type fly displays opposing taste responses to low and high concentrations of acid: low concentrations are attractive, whereas high concentrations are aversive. Thus, the hedonic valence of acid taste is closely associated with the concentration of acid that the animals detect. The bidirectional valence of sour taste is reminiscent of salty taste, which is also dependent on salt concentrations^22^. Given these findings, a key question arises as to how the animals discern low- from high-acid foods. Our electrophysiology analyses of different groups of taste sensilla provide an important clue to this question. We found that flies mainly use L- and S-type sensilla to perceive low and high concentrations of acid, respectively. The L-type sensilla mediate an attractive pathway, whereas the S-type sensilla operate an aversive pathway for the response to acids. We postulate that acid-taste signals relayed by low- and high-acid GRNs antagonize each other in the brain, where feeding decisions are made. When the fly encounters low-acid foods, the attractive pathway mediated by low-acid GRNs dominates the aversive pathway mediated by the high-acid GRNs, driving the animal to choose the low-acid food. Conversely, when the animal encounters high-acid foods, the aversive pathway dominates the attractive pathway, leading to the avoidance of high-acid foods.

In support of this hypothesis, our genetic analysis reveals that *otopla* is specifically required for the attractive rather than aversive acid-taste response. Further, we found that *otopla* is mostly expressed in the GRNs housed within the L-type rather than the S-type sensilla. In addition, recent work shows that a member of the ionotropic receptor (IR) family, *Ir7a*, is selectively required for the repulsive response to high concentrations of acetic acid in *Drosophila*^10^. However, flies lacking *Ir7a* show normal attractive feeding responses to low concentrations of acetic acid. That study, combined with our present study on *otopla*, provides substantial evidence to support our model that the attractive and aversive pathways for acid sensation are segregated in the peripheral taste organ.

These findings lay the foundation for a more detailed analysis of the genetic program and neural circuit involved in sour-taste perception. In mammals, type III TRCs are mainly responsible for sour-taste sensation^1,2^. Nevertheless, whether distinct subgroups of type III TRCs in the taste bud selectively respond to low or high acid remains an open question. Given the conservation of acid-taste sensation between flies and mammals, the acid-taste coding mechanism identified in the fly will inform the investigation of sour-taste coding in mammals, including humans.

Using multiple lines of evidence, our studies lead to the identification of OtopLa as a long-sought taste receptor for acid in *Drosophila*. First, our genetic analyses show that OtopLa is both necessary and sufficient to orchestrate the attractive response to foods containing low concentrations of acid. In addition, loss of *otopla* has no effect on sweet, bitter, or salty tastes. Second, our cell biological studies reveal that OtopLa is expressed in a group of GRNs different from sweet, bitter, and salty GRNs. Moreover, OtopLa proteins selectively reside in the distal portion of the dendrite, the forefront of the GRN responsible for detecting taste substances presented from the food environment. Last but not least, our patch-clamp recordings reveal that OtopLa functions as a proton channel and can be directly activated by protons. Collectively, our work establishes OtopLa as a bona fide receptor that is dedicated to the attractive taste sensation of acids in *Drosophila*. Recent studies in mice show that the Otop1 proton channel is both necessary and sufficient for sour-taste transduction^14,15^. In light of these discoveries in flies and mice, we conclude that the Otop family is an evolutionarily conserved proton channel dedicated to taste the sensation of acids in both insects and mammals. Over the past two decades, various types of taste receptors, including sweet, bitter, and salty taste receptors, have been identified and functionally characterized in both invertebrates and vertebrates^2,13,38,39^. Although insects and mammals exhibit a striking homology in taste responses, the molecular identities of their sweet, bitter, and salty taste receptors appear to be distantly related to each other. Consequently, there is a long-held view in the chemoreception field that taste receptors for insects and mammals are evolutionarily distant from each other. Here, our discovery in the fly acid-taste sensation has overturned this notion. To the best of our knowledge, the Otop family represents the first class of taste receptors that is functionally conserved between insects and mammals. From an evolutionary perspective, we propose that Otop is a well-conserved proton channel family involved in acid-taste sensation throughout the animal kingdom. Thus, further research is needed to explore the gustatory role of the Otop family in other animal species, including humans.

We found that *otopla* is necessary for the attractive taste sensation of both the strong acid HCl and the weak acids citric acid and malic acid. Psychophysical studies in human subjects report that weak acid usually tastes more sour than strong acid at the same pH^40^, implying that, in addition to protons, the undissociated weak acid molecules may also elicit sourness. Our studies demonstrate that the proton channel OtopLa is broadly required for the taste sensation of both strong and weak acids. Therefore, we propose that the taste response to acids orchestrated by OtopLa mainly results from the gustatory stimuli of protons that are dissociated from either strong or weak acids. In addition, several members of the fly IR family are involved in taste responses to carbonation^41,42^ and acetic acid^10^. As there has been no evidence showing that the IRs form a proton channel, the IRs are likely to be narrowly tuned to the specific structures of weak acids rather than to protons. Collectively, the fly may use different taste transduction pathways to perceive various acid molecules present in the environment.

In conclusion, given the significant conservation of taste receptors for acid between flies and mammals, our fly model will significantly advance our understanding of the acid-taste sensation in other animals, including humans.

## Methods

### Materials and reagents

#### Fly strains

The fly lines used in this study are as follows: *otopla*^*1*^, wild-type flies (*w*^*1118*^), *UAS-otopla*, *UAS-otoplaE638A*, *UAS-otopla RNAi* (RRID_VDRC_104973), *UAS-otoplb RNAi* (RRID_VDRC_101936), *UAS-otoplc RNAi* (RRID_VDRC_108591), *Otopla-Gal4*, *Gr5a-LexA-VP16*^34^, *Gr5a-Gal4* (RRID_BDSC_57592), *Gr66a-LexA-VP16*^35^, *Gr66a-Gal4* (RRID_BDSC_57670), *poxn-Gal4* (RRID_BDSC_66685), *Ir76b-QF*^22^, *UAS-mCD8::GFP* (RRID_BDSC_5137), *UAS-mCD8::DsRed* (RRID_BDSC_27399), *QUAS-mtdTomato*^36^ (RRID_BDSC_30005), and *LexOP-mCherry-HA*^43^ (RRID_BDSC_52271).

#### Antibodies

The antibodies used in this study are as follows: rabbit anti-GFP polyclonal antibody (Thermo Fisher, catalog number A-11122, RRID_AB_221569), mouse anti-nc82 monoclonal antibody (Developmental Studies Hybridoma Bank (DSHB), catalog number nc82, RRID_ AB_528108), mouse anti-c-Myc monoclonal antibody (clone 9E10; Thermo Fisher, catalog number MA1-980, RRID_AB_558470), rabbit anti-OtopLa polyclonal antibody (generated in this study), mouse anti-mCherry monoclonal antibody (DSHB, catalog number DSHB-mCherry-3A11, RRID_AB_ 2617340), goat anti-rabbit Alexa Fluor 488 (Jackson ImmunoResearch, catalog number 111-585-003, RRID_AB_2338059), goat anti-mouse Alexa Fluor 594 (Jackson ImmunoResearch, catalog number 115-545-003, RRID_AB_2338840), and donkey anti-mouse Alexa Fluor 594 (Jackson ImmunoResearch, catalog number 715-585-150, RRID_AB_ 2340854).

### Experimental methods

#### Generating the *otopla*^*1*^ mutant

We took advantage of CRISPR/Cas9-mediated gene editing to knock out a DNA fragment including the TM6 region of the OtopLa protein (Supplementary Fig. 3a–c), thereby rendering the OtopLa channel dysfunctional. To do this, we designed two gRNAs flanking the knocked out region (Supplementary Table 1):

gRNA1: 5′-GGC CCA GAT CGG TGT ATG TCT GG-3′;

gRNA2: 5′-GGA GGT GAT GTT ATC GCG GCG GG-3′.

The two gRNA-targeting sites, which are ~300 bp apart within the same exon (Supplementary Fig. 3a), are predicted to have little off-target activity. Complementary oligonucleotides for these two gRNAs were purchased from Integrated DNA Technologies (Coralville, IA, USA), annealed, and ligated into the *pU6-BbsI-chiRNA* plasmid (RRID: Addgene_45946) to generate two separate gRNA plasmids.

To induce deletion by non-homologous end joining, a mixture of these two gRNA plasmids at 1 : 1 ratio was injected into embryos of a fly strain expressing the Cas9 protein under the control of the *vas* promoter (*vas-Cas9*) (Bloomington  *Drosophila* Stock Center (BDSC), RRID_BDSC_56552) (BestGene, Inc., Chino Hills, CA, USA). We separated viable recipient flies and individually mated them with wild-type flies. We screened the offspring of single mating for deletion mutants using PCR assays. In brief, we extracted genomic DNA from the fly body with a nucleotide extraction kit according to the manufacturer’s protocol (G-spin™ Total DNA Extraction Kit, iNtRON Biotechnology, catalog number 17046). We then amplified a DNA fragment containing the desired deletion by PCR with the following primers (Supplementary Table 1):

Forward primer: 5′-CTA CTA TGG CCC GCA AGC TG-3′;

Reverse primer: 5′-TGC AAA GTA CAC AAC GAA CGA G-3′.

Flies harboring the *otopla* deletion yielded a shorter PCR product than wild-type flies (Supplementary Fig. 3b), which we used to select for mutants. To determine the exact length and sequence of the deleted DNA fragment, PCR products from wild-type and mutant lines were purified and then sequenced. We further outcrossed validated deletion-mutant flies to wild-type flies for five generations, to eliminate potential background mutations. Finally, we balanced the mutant to establish a stable *otopla*^*1*^ mutant line.

#### Generating the *otopla* promoter *Gal4* line

To make a promoter *Gal4* line for *otopla*, we performed PCR to amplify a 3015 bp promoter region located upstream of the *otopla* coding region (Fig. 2a), using the following primer pairs:

Forward primer: 5′-A TAC ATA CTA GAA TTC GAT CTC AAC TGC TCC TCT CCT CTC-3′;

Reverse primer: 5′-T TTG CTT ACG GGA TCC TCC TTT CCC CTC GCT CAG CA-3′.

In short, we extracted genomic DNA from wild-type fly bodies using the G-spin™ Total DNA Extraction Kit. After confirming the cloned promoter sequence, we ligated the promoter fragment between EcoRI and BamHI restriction sites in the *pCaSpeR-Gal4* vector using In-Fusion cloning (In-Fusion® HD Cloning Kit, Clontech, catalog number 639650). The *otopla* promoter construct’s fidelity was verified by sequencing.

To make an *otopla-Gal4* transgenic line, germline-mediated transformation was performed by injecting the *pCaSpeR-otopla-Gal4* plasmid into wild-type fly embryos (BestGene). We screened viable recipient lines using red eyes as a positive marker. We outcrossed red-eyed transgenic flies to wild-type flies for five generations, to eliminate any potential mutations in their genetic backgrounds. We then determined their chromosomal insertion sites to establish stable *otopla-Gal4* transgenic lines.

#### Generating the *UAS-otopla* line

To generate a *UAS-otopla* transgenic line, we first amplified the full cDNA of *otopla* (isoform A) from a full-length sequenced cDNA clone obtained from the *Drosophila* Genomics Resource Center (clone RE56564) using the following primer pairs:

Forward: 5′-C GCT CAT ATG GAA TTC ATG GGC GGC GGT GAA GTG AAG-3′;

Reverse: 5′-TAGAGGT ACC CTC GAG TTA CTC CAG ACG TGC CTT GTA GGT G-3′.

We ligated the PCR cloned fragment into the *pUAST* vector using the EcoR1 and XhoI cloning sites at the upstream and downstream ends, respectively. The resulting plasmid was injected into wild-type flies for P-element-mediated transformation (BestGene). We identified transformants by the red-eye phenotype and outcrossed them to wild-type flies for five generations to remove any genetic background mutations.

#### Generating the *UAS-otoplaE638A* line

We predicted the transmembrane topology of OtopLa protein using RHYTHM (http://proteinformatics.de/rhythm). We selected the E638 residue of OtopLa to make an E638A point mutation by site mutagenesis (QuikChange II Site-Directed Mutagenesis Kit, Agilent, catalog number 200523) using the following primers:

Forward: 5′-CGC CCC GAT CAG CGC ATA C(T → G)C GAT GAT GAA CGG A-3′;

Reverse: 5′-T CCG TTC ATC ATC G(A → C)G TAT GCG CTG ATC GGG GCG-3′.

Mutations were subsequently confirmed by DNA sequencing. To create a *UAS-otoplaE638A* line, we used Phusion® High-Fidelity DNA Polymerase (New England Biolabs, catalog number M0530) to faithfully clone the *otoplaE638A* cDNA and insert it between the EcoRI and BamHI restriction sites in a new *pUAST* vector. We then used this plasmid for germline-mediated transformation (BestGene). We established the stable *UAS-otoplaE638A* transgenic lines after backcrossing them to wild-type flies for five generations.

#### Generating constructs for cell transfection

We chose *pcDNA3-EGFP* (RRID_Addgene_13031) and *pCS2+MT* as expression vectors in HEK293 cells. To monitor OtopLa expression in HEK293 cells, we tagged OtopLa with either enhanced GFP in the *pCDNA3* vector or Myc in the *pCS2+MT* vector. To make the *pCDNA3* construct, we amplified the full-length cDNAs of *otopla* and *otoplaE638A* by PCR using their respective *pUAS* constructs as templates and the following primers:

Forward: 5′-T TGC GGC CGC GAA TTC ATG GGC GGC GGT GAA GTG AAG-3′;

Reverse: 5′-T GGT GGC GAT GGA TCC CTC CAG ACG TGC CTT GTA GGT G-3′.

We subsequently ligated the cDNA fragments for *otopla* and *otoplaE628A* into the *pcDNA3* vector at the EcoR1 and BamH1 cloning sites. The same method was used to generate the *pCS2+MT* construct, using the following primers:

Forward: 5′-A GAG GAC TTG AAT TCA ATG GGC GGC GGT GAA GTG AAG-3′;

Reverse: 5′-G TTC TAG AGG CTC GAG TTA CTC CAG ACG TGC CTT GTA GGT G-3′.

The PCR cloned fragments were subsequently ligated into the *pCS2+MT* vector at the EcoRI and XhoI cloning sites. Sequence fidelity of all constructs was confirmed by DNA sequencing.

#### Generating OtopLa antibodies

To generate antibodies against OtopLa, we chose a 45-amino-acid segment (residues 547–591), located in the large extracellular domain between TM5 and TM6 (Supplementary Fig. 4a). This peptide is present in all OtopLa isoforms and shares little sequence homology with other fly proteins. We amplified the cDNA fragment encoding the 45-amino-acid peptide by PCR using the following primers:

Forward: 5′-G GGG CCC CTG GGA TCC GCC CAC TCG ATT CGT CAG C-3′;

Reverse: 5′-G ATG CGG CCG CTC GAG TTA CAC ATT GTC GCT CTT GTA CAC AT-3′.

Next, we subcloned the cDNA fragment into the EcoRI and XhoI sites of the GST fusion vector *pGEX6-P-1* (Cytiva, catalog number 28954648). We induced the expression of GST-OtopLa fusion proteins with isopropyl β-d-1-thiogalactopyranoside (LabScientific, catalog number I-555) in BL21 competent *Escherichia coli* cells (New England Biolabs, catalog number C2530H). We then purified the GST-OtopLa fusion protein from bacterial cell lysates using GSTrap affinity columns (Cytiva, catalog number 28401745), followed by cleaving the GST tag via PreScission protease (Cytiva, catalog number 27084301). The purified proteins were used as antigens to raise polyclonal antibodies in a rabbit host (Pacific Immunology, Ramona, CA USA).

#### Two-way feeding assay

To perform two-way choice behavioral assays, we used a Petri dish-based paradigm. A 35 mm-diameter plastic Petri dish was bisected evenly by a plastic divider, creating two separate compartments that can be filled with food. One half of the dish contained 1% agarose (Denville Scientific, catalog number GR140-500) and 2 mM sucrose (Sigma-Aldrich, catalog number S7903); the other half contained 2 mM sucrose with the addition of a given concentration of acid. Acids used in this study are HCl (Sigma-Aldrich, catalog number 320331), citric acid (Sigma-Aldrich, catalog number 251275), and malic acid (Sigma-Aldrich, catalog number 240176). Each of these foods was mixed with either red (sulforhodamine, Sigma-Aldrich, catalog number S9012) or blue (Brilliant Blue FCF, FUJIFILM Wako Chemicals USA Co., catalog number 3844-45-9) dye. After wet-starving the 2- to 4-day-old adult flies for 24 h, we transferred the flies into the dish containing two different food options and allowed them to select the preferred food source at room temperature for 90 min in dark conditions. We visually assessed the color of their abdomens under a dissection scope to generate a preference index (PI):

PI = (*N*_red_ − *N*_blue_)/(*N*_red_ + *N*_blue_ + *N*_purple_) if the acid was mixed into the red food or

PI = (*N*_blue_ − *N*_red_)/(*N*_red_ + *N*_blue_ + *N*_purple_) if the acid was mixed into the blue food.

Here, *N*_red_ equals the number of flies colored red from eating red food, *N*_blue_ is the number of flies colored blue from eating blue food, and *N*_purple_ is the number of flies colored purple from eating both. A PI of 0 indicates no preference, whereas a PI of ±1.0 indicates a complete preference for one food or the other. Each replicate used ~70 animals, and we performed at least 3 replicates of each color option. Data for the behavioral assays were collected under double-blind conditions, wherein the scorer did not know the identity of each sample being scored.

#### PER assay

Prior to the PER assay, we wet-starved 2- to 4-day-old adult flies for 12–24 h at room temperature. Next, we gently slid a pre-starved fly through a 20 μl pipette tip and trimmed off the tip of the pipette with a razor blade so that only the fly head and proboscis were exposed, leaving other parts of the body such as legs and wings still restrained inside the pipette tube. After affixing the fly preparation under a dissection microscope (Amscope, ×10) using modeling clay, we applied a drop of test solution (2–5 μl), such as sucrose or sucrose mixed with HCl, citric acid, or malic acid, to the fly proboscis and noted the subsequent PER response, if any. To ensure the fly remained viable throughout the experiment, we stimulated the fly with a drop of sucrose (200 mM) immediately after the PER assay; only data from flies showing obvious PERs in response to 200 mM sucrose were subject to further statistical analyses. In each trial, we analyzed at least ten flies, and the PER percentage was calculated as the percentage of flies showing proboscis extensions in response to taste stimuli.

#### Cell culture and transfection

We cultured HEK293 cells (ATCC, catalog number ATCC® CRL-2828^™^, RRID_CVCL_0045) in 35 mm Petri dishes (Fisher Scientific, catalog number 12-556-000) with a medium composed of Opti-MEM^TM^ Medium (Gibco^TM^, Opti-MEM^TM^ Reduced Serum Medium, Thermo Fisher Scientific, catalog number 51985034), 10% fetal bovine serum (Gibco^TM^, Thermo Fisher Scientific, catalog number 16140063), and 100 U/mL penicillin–streptomycin (Gibco^TM^, Thermo Fisher Scientific, catalog number 15140122). We maintained the cells in a tissue culture incubator at 37 °C with 5% CO_2_. Cells were passaged once a week, and the culture medium was replaced every 3 days.

We co-transfected pcDNA3-OtopLa or pcDNA3-OtopLaE638A together with pcDNA3-GFP (5 : 1) into HEK293 cells using Lipofectamine^TM^ 3000 transfection reagent (Invitrogen^TM^, Thermo Fisher Scientific, catalog number L3000008). Cells were treated with 0.25% trypsin (Thermo Fisher Scientific, catalog number 15090046) 24 h after transfection and were subsequently plated onto coverslips coated with poly-l-lysine (Sigma-Aldrich, catalog number P4707) for the patch-clamp recording. The same procedure was used to co-transfect pCS2+MT-OtopLa or pCS2+MT-OtopLaE638A.

#### Whole-cell patch-clamp electrophysiology

We performed whole-cell patch-clamp recordings using an Axopatch 200B amplifier (Molecular Devices, San Jose, CA, USA) and an Axon^TM^ Digidata 1550B digitizer (Molecular Devices). Data were digitized at 10 kHz with a 2 kHz low-pass filter. We used the Clampex 10.6 software (Axon Instruments, CA, USA) for data acquisition, and data were analyzed using Clampfit 10.6 software (Axon Instruments). Patch pipettes (6–10 MΩ) were pulled from borosilicate glass capillaries (Sutter Instrument Co., Novato, CA, USA, catalog number BF150-86-10) with the P-1000 micropipette puller (Sutter Instrument, Co.). The internal pipette solution contained 120 mM Cs-methanesulfonate, 15 mM CsCl, 2 mM Mg-ATP, 5 mM EGTA, 1 mM CaCl_2_, and 10 mM HEPES (pH 7.3 with Tris; 290 mOsm). The extracellular solutions bathing the cells contained 160 mM NMDG-gluconate, 2 mM CaCl_2_, and 10 mM HEPES (pH 7.3; 320 mOsm). We carried out series resistances and cell capacitance compensation prior to recording. We adjusted the acidification of extracellular solutions with HCl, and acid stimulus was locally applied to the cell at a very close range. In the ion replacement experiments, we tested different isosmotic bath solutions (160 mM), including NaCl, KCl, LiCl, NMDG-Cl, and NMDG-gluconate. After the whole-cell configuration was achieved, the membrane potential was held at −60 mV. The recordings were performed only in those with gigaohm seals.

#### Tip recording

We chose 2- to 4-day-old adult flies for tip recordings. After anesthetizing the fly on ice, we inserted a sharp glass pipette (World Precision Instruments, catalog number 1B150F-3), which was pulled by a P-1000 micropipette puller (Sutter Instrument Co., catalog number P-1000), at the fly thorax and gently pushed the sharp pipette through the neck to eventually reach the labellum. To facilitate our recordings, we finely adjusted the position of the glass pipette to get the fly labellum fully extended and secured. We performed tip recordings in a rig composed of a headstage (Syntech), a taste probe (Syntech), a four-channel data acquisition controller (Syntech), and a computer system. The spike signals are sampled at a rate of 12,000 and filtered at 1 kHz. The spike train was analyzed by the Autospike software (Syntech).

For all tip recordings, the solution in the reference pipette contained 130 mM NaCl, 2 mM KCl, 2 mM CaCl_2_, 5 mM HEPES, and 40 mM sucrose (pH 7.2). Regarding the solutions in the recording pipette, we chose 1 mM KCl to record taste responses to caffeine (Sigma-Aldrich, catalog number C0750), NaCl, HCl, citric acid, and malic acid, whereas we used 30 mM tricholine citrate (Sigma-Aldrich, catalog number T0252) to assess taste responses to sucrose.

#### Immunocytochemistry and confocal microscopy

We dissected proboscis and brain tissues from 2- to 4-day-old adult flies in phosphate-buffered saline (PBS). After fixing the isolated tissues in 4% paraformaldehyde (Electron Microscopy Sciences, catalog number 15710), we blocked the samples with 0.5% normal goat serum (Cell Signaling Technology, catalog number 5425 S) in 1× PBS and then incubated them with primary antibodies in 1× PBST buffer (PBS + 0.2% Triton™ X-100; Fisher Scientific, catalog number BP151100) at 4 °C for 24–48 h. The primary antibodies used in this study include rabbit anti-GFP (1 : 400; Invitrogen, catalog number A-11122), mouse anti-nc82 (1 : 40; DSHB, catalog number nc82), mouse anti-mCherry (1 : 20; DSHB, catalog number 3A11), rabbit anti-OtopLa (1 : 400), and mouse anti-GFP (1 : 200; Invitrogen, catalog number A11120). After extensively washing the samples with PBST, we incubated them with secondary antibodies at 4 °C for 24 h. The secondary antibodies we used in this study are goat anti-rabbit Alexa Fluor 488 (1 : 200; Jackson ImmunoResearch, catalog number 111-585-003), goat anti-mouse Alexa Fluor 594 (1 : 200; Jackson ImmunoResearch, catalog number 115-545-003), and donkey anti-mouse Alexa Fluor 594 (1 : 200; Jackson ImmunoResearch, catalog number 715-585-150). Finally, we mounted the fly proboscis or brain samples on a glass slide with the VECTASHIELD antifade mounting medium (Vector Laboratories, catalog number H-1900).

To perform immunocytochemistry of HEK293 cells expressing Myc-tagged OtopLa or OtopLaE638A, we placed a thin coverslip (Fisher Scientific, catalog number 12542BP) in the cell culture dish and allowed cells to grow on the coverslip surface for 24 h. After transfection, the coverslip was fixed with 4% paraformaldehyde at room temperature for 30 min. After washing the coverslip with 1× PBS (non-permeabilized) or 1× PBST (PBS + 0.2% Triton™ X-100) (permeabilized), we incubated it with primary antibodies suspended in 1× PBS (non-permeabilized) or 1× PBST (permeabilized), including mouse anti-Myc (1 : 50) and rabbit anti-OtopLa (1 : 1000), at room temperature for 2 h. After washing the coverslip with 1× PBS (non-permeabilized) or 1× PBST (permeabilized) to remove unbound antibodies, we incubated it with secondary antibodies diluted in 1× PBS (non-permeabilized) or 1× PBST (permeabilized), including goat anti-rabbit Alexa Fluor 488 (1 : 200; Jackson ImmunoResearch, catalog number 111-585-003) and donkey anti-mouse Alexa Fluor 594 (1 : 200; Jackson ImmunoResearch, catalog number 715-585-150), for another 2 h. Finally, the coverslip was mounted on a clear glass slide.

Using a Zeiss 710 confocal microscope (Cell and Developmental Biology Microscopy Core, University of Pennsylvania), we collected single and Z-stack images from the fly proboscis and brain, as well as the transfected HEK293 cells. The acquired confocal images were analyzed using Zeiss 710 native imaging software or ImageJ.

### Statistical analyses

We chose the unpaired, two-tailed Student’s *t*-test if only two samples were involved in the analysis. For three or more samples, we used the one-way analysis of variance (ANOVA) test followed by Tukey’s post hoc analysis. Statistical significance was set at *p* < 0.05 threshold. In the two-way feeding assay, *n* represents the number of trials. In the PER assay, *n* represents the number of trials. For tip recordings, *n* represents the number of flies. For patch-clamp analyses, *n* represents the number of cells.

### Reporting summary

Further information on research design is available in the Nature Research Reporting Summary linked to this article.

## Supplementary information

Supplementary Information

Reporting Summary

## Data Availability

All data are available in the manuscript and Supplementary Information, or are available upon request from the corresponding author.
