# Supplementary material for: Molecular and cellular basis of acid taste sensation in Drosophila
Source: Nat Commun. 2021 Jun 17;12:3730. doi: 10.1038/s41467-021-23490-5 (PMC8211824; doi:10.1038/s41467-021-23490-5)
Supplement: Supplementary file 1 — Supplementary Information [file 41467_2021_23490_MOESM1_ESM.pdf]

Supplementary Fig. 1

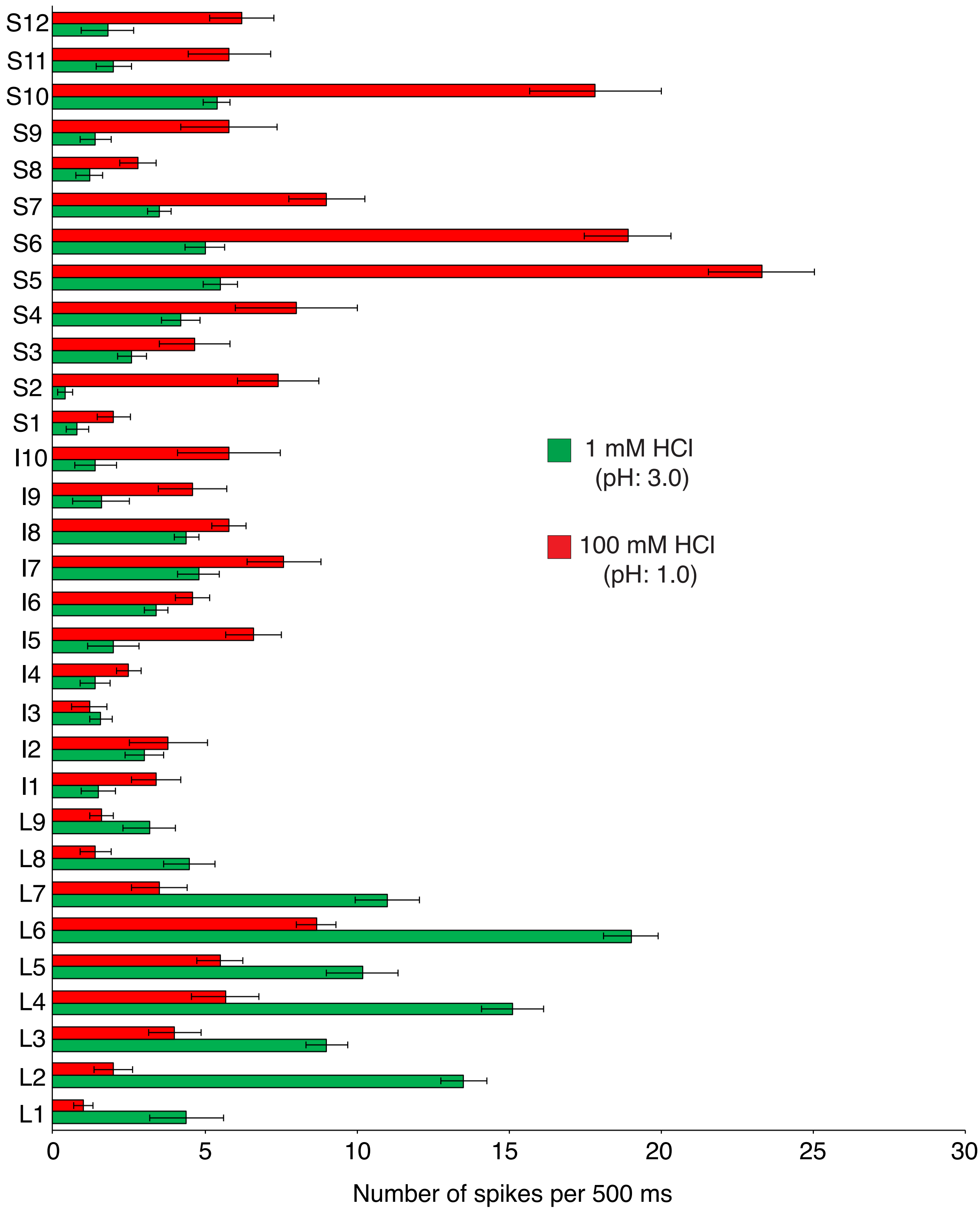

Supplementary Fig. 1. Electrophysiological responses to low (1 mM) and high (100 mM) concentrations of HCl for 9 L-type, 10 I-type, and 12 S-type sensilla in the wild-type fly. Data are presented as mean ± SEM, *n* = 11 animals.

Supplementary Fig. 2

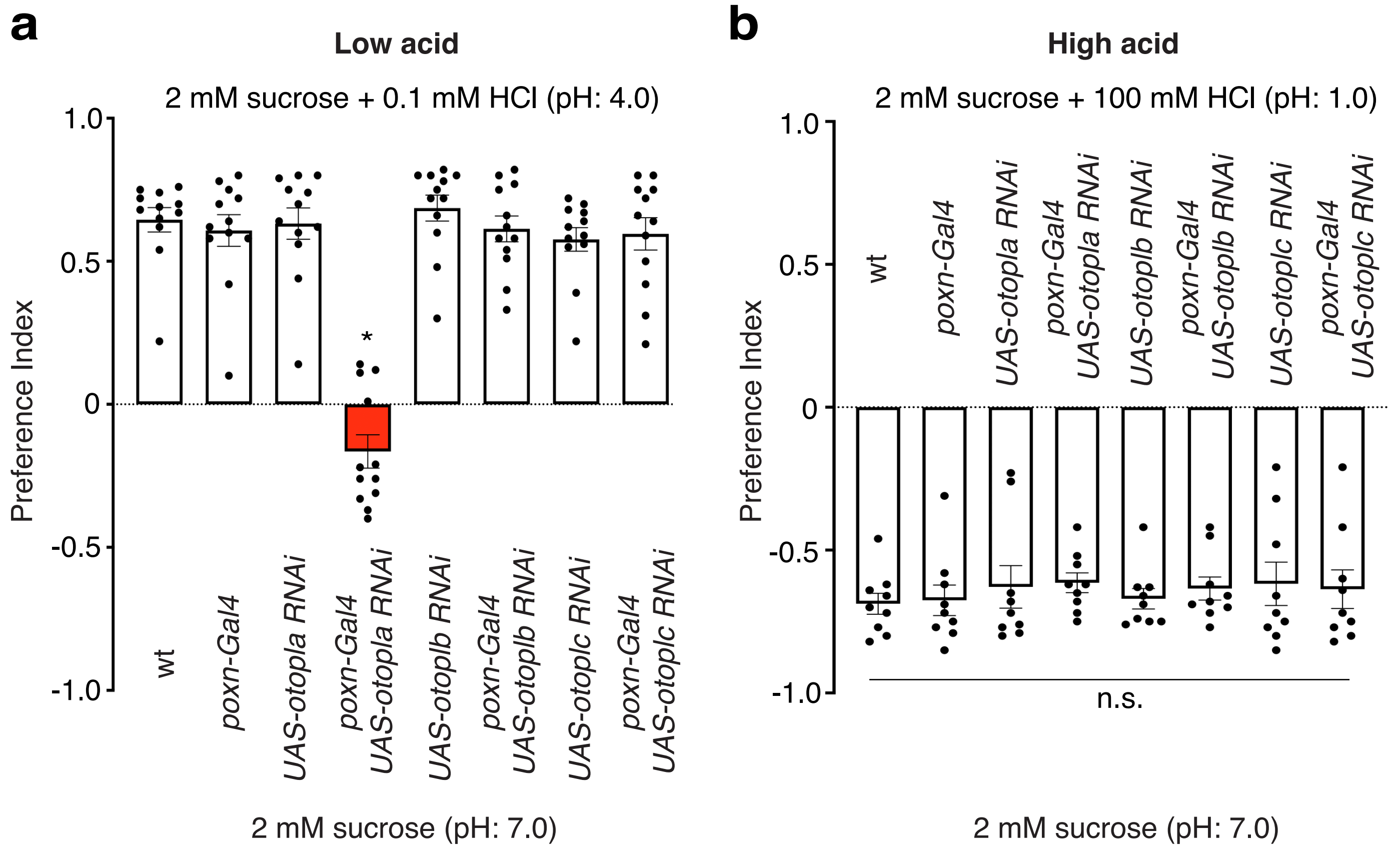

Supplementary Fig. 2. Feeding responses to low acid and high acid for *otopla*, *otoplb*, and *otoplc* knockdown flies. We used the *poxn-Gal4* to selectively knock down three fly *otop* genes, including *otopla*, *otoplb*, and *otoplc,* in the GRNs. The resulting *poxn-gal4/UAS-otopla RNAi*, *poxn-gal4/UAS-otoplb RNAi*, and *poxn-gal4/UAS-otoplc RNAi* flies, as well as wild type (wt) flies were allowed to choose between neutral foods containing 2 mM sucrose and mildly acidic foods containing 2 mM sucrose and 0.1 mM HCl (**a**), or highly acidic foods containing 2 mM sucrose and 100 mM HCl (**b**). Data are presented as mean ± SEM. n = 12 trials, *$p < 0.0001$, one-way ANOVA tests (**a**). n = 9 trials, n.s., not significant, one-way ANOVA tests (**b**).

Supplementary Fig. 3

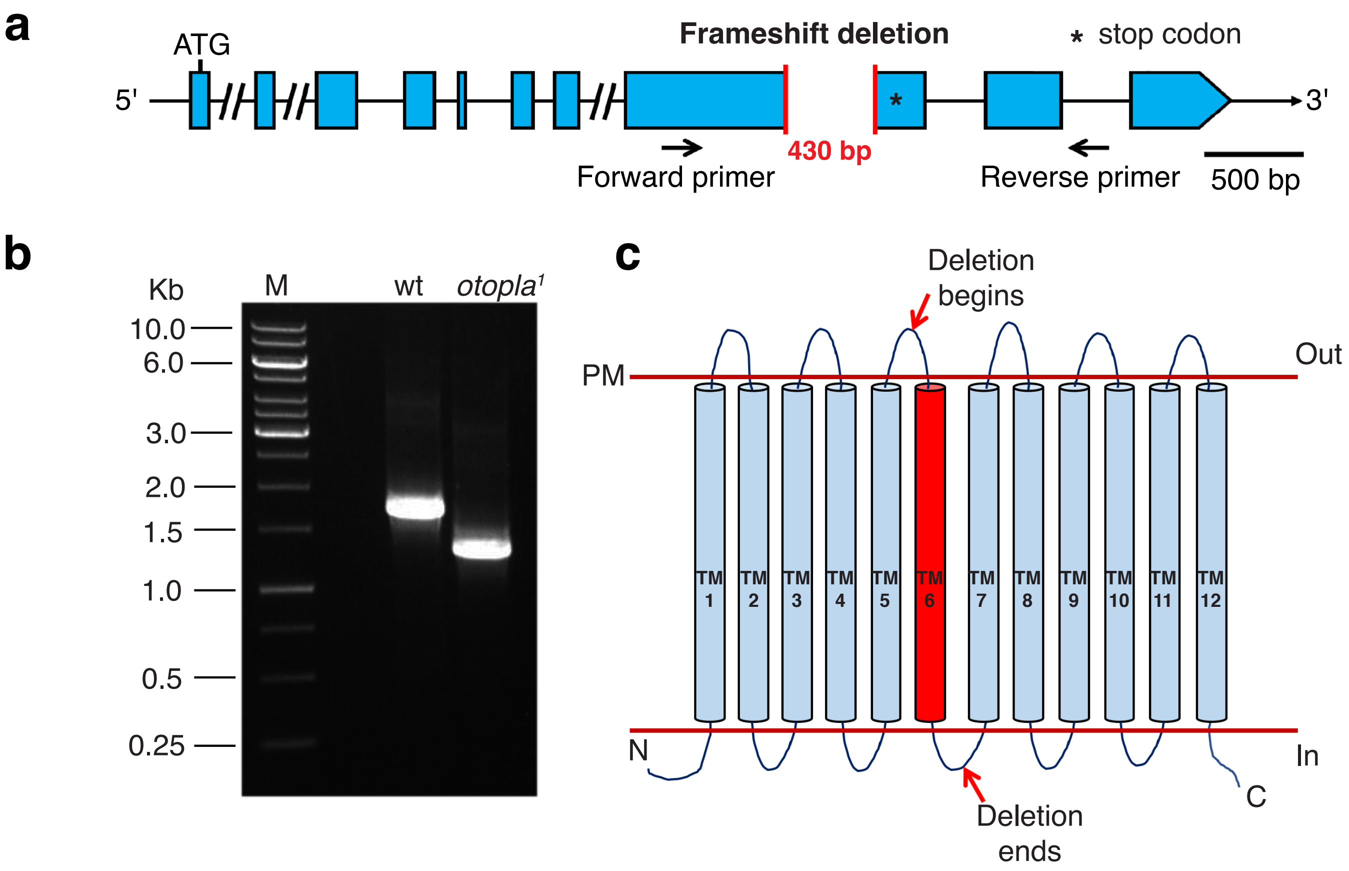

Supplementary Fig. 3. Molecular lesions associated with the *otopla*[1] mutant. (**a**) The deleted exon region and the primer pairs used to screen for the deletion mutant. (**b**) PCR analysis of the deletion, with wild type (wt) as a positive control. The PCR products were resolved through DNA gel electrophoresis. A loss of 430 nucleotides was found in the *otopla*[1] mutant as compared to wt. (**c**) A predicted topology of the fly OtopLa protein comprising 12 transmembrane segments. Red arrows mark the ablated protein region containing the 6th transmembrane segment (red) in the *otopla*[1] mutant. Moreover, the frameshift deletion generates a premature stop codon within the partially deleted exon, ablating all transmembrane segments downstream from the deletion. PM: plasma membrane.

Supplementary Fig. 4

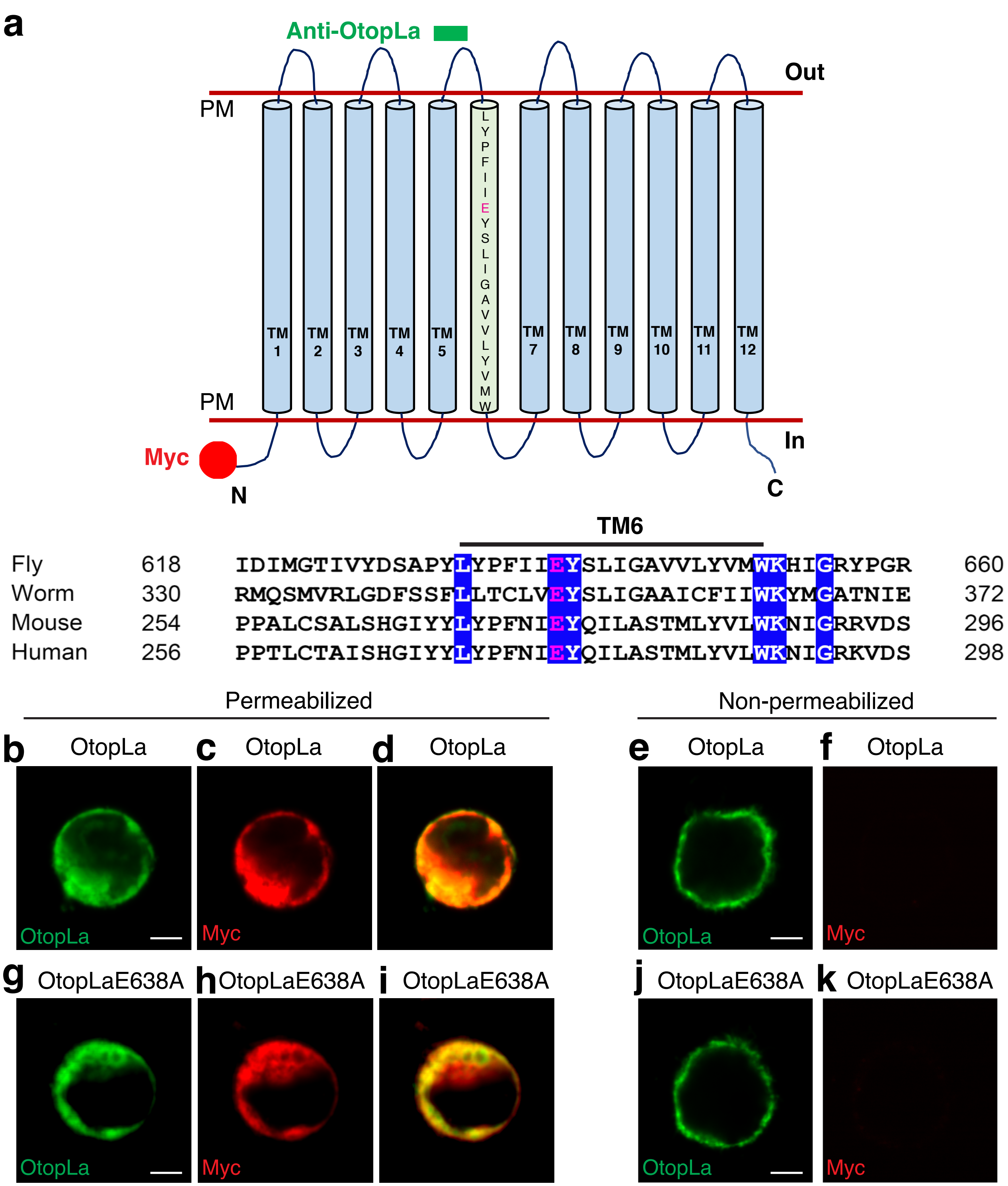

Supplementary Fig. 4. Immunocytochemical analyses of the HEK293 cells expressing OtopLa or OtopLaE638A. (**a**) Top: a predicted topology of the fly OtopLa showing a stretch of 20 amino acid residues that form the 6th transmembrane (TM6) segment. PM: plasma membrane. Bottom: a multiple-sequence alignment of the TM6 segments of Otop1 protein orthologs from flies (dOtopLa), worms (cOtop1), mice (mOtop1), and humans (hOtop1). The glutamate (E) residue, which is highly conserved across different species, is highlighted in pink. The other conserved amino acid residues are highlighted in blue. (**b – f**) Immunocytochemical analyses of HEK293 cells expressing wild-type OtopLa tagged with Myc, with and without detergent. The cells were double-labeled with anti-Myc and anti-OtopLa. (**g – k**) Immunostaining of the HEK293 cells expressing Otop-LaE638A tagged with Myc, with and without detergent. The cells were costained with anti-Myc and anti-OtopLa. All immunocytochemical experiments were repeated at least three times with similar results. Scale bars: 5 µm.

Supplementary Fig. 5

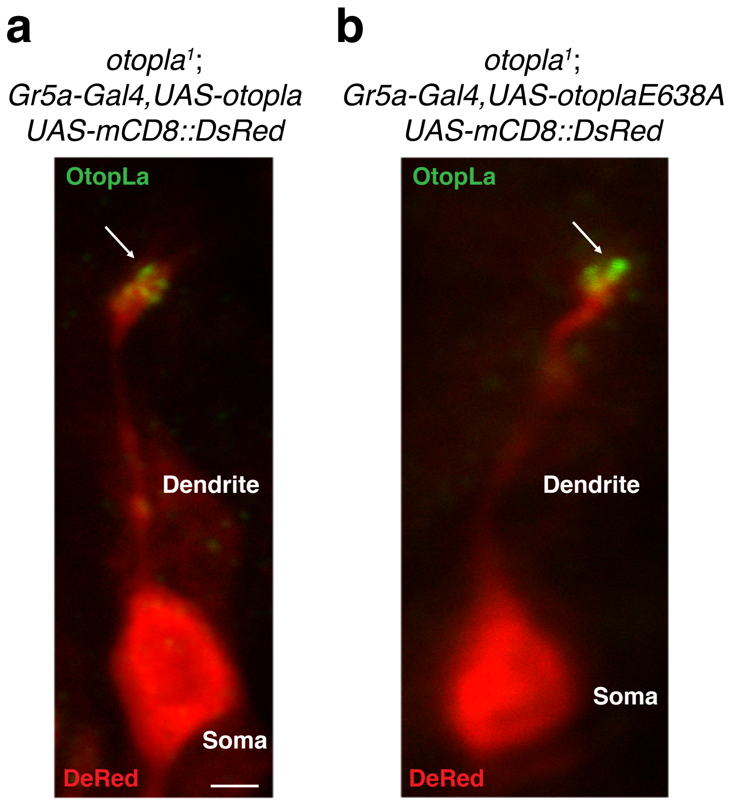

Supplementary Fig. 5. Localization of wild-type OtopLa and OtopLaE638A mutant misexpressed in the sweet GRNs. We misexpressed wild-type OtopLa (**a**) and OtopLaE638A mutant (**b**) at the sweet GRNs in the *otopla*^{1} mutant background. In the same animal, we co-expressed a membrane-tethered *UAS-mcD8::DsRed* reporter to highlight the soma and dendrite of GRNs. Arrows point to the tip of the dendrite. All immunocytochemical experiments were repeated at least three times with similar results. Scale bar: 2 μm.

Supplementary Fig. 6

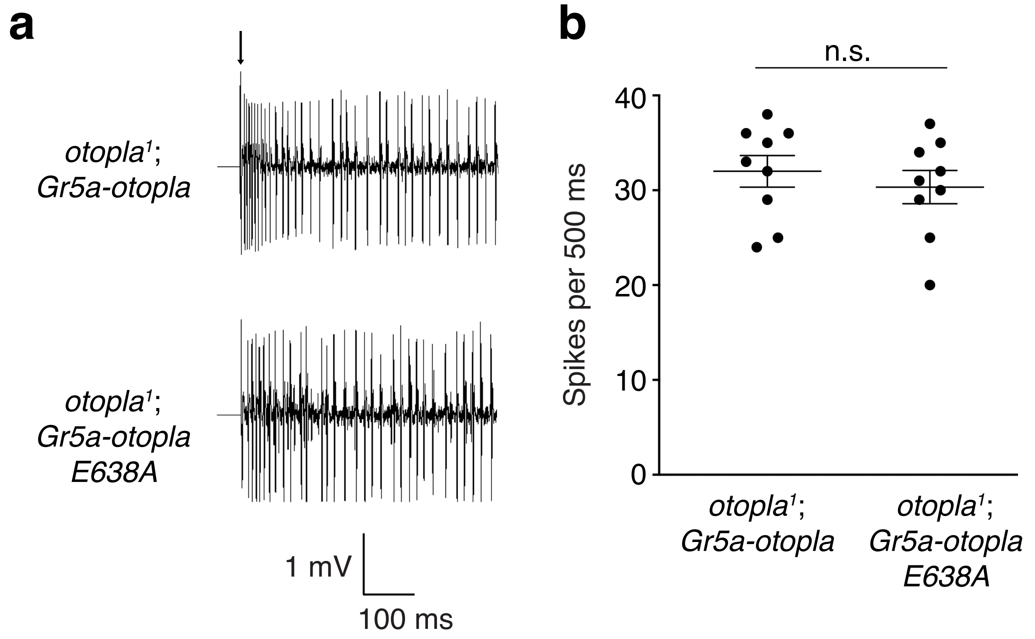

Supplementary Fig. 6. Electrophysiological responses to sucrose in sweet GRNs misexpressing OtopLa or OtopLaE638A. (**a**) Representative spike trains fired by the L8 sensilla in response to 50 mM sucrose in *otopla[1]* mutant flies misexpressing either OtopLa or OtopLaE638A in sweet GRNs. The arrow indicates the onset of taste stimuli. (**b**) Statistical analysis of the firing frequency in OtopLa or OtopLaE638A misexpressing flies. $n = 9$ animals. Data are presented as mean ± SEM. n.s., not significant, unpaired two-tailed *t*-tests.

Supplementary Table 1

Supplementary Table 1. Sequences of primers used in this study

Vector	Construct	Primers
pU6-BbsI-chiRNA	*otopla* gRNA1	Sense: 5'-GGCCCAGATCGGTGTATGTCTGG-3'<br><br>Antisense: 5'-CCAGACATACACCGATCTGGGCC-3'
pU6-BbsI-chiRNA	*otopla* gRNA2	Sense: 5'-GGAGGTGATGTTATCGCGGCGGG-3'<br><br>Antisense: 5'-CCCGCCGCGATAACATCACCTCC-3'
N/A	$otopla^{1}$ mutant screening	Forward: 5'-CTACTATGGCCCGCAAGCTG-3'<br><br>Reverse: 5'-TGCAAAGTACACAACGAACGAG-3'
pCaSpeR	*otopla-Gal4*	Forward: 5'-ATACATACTAGAATTCGATCTCAACTGCTCCTCTCCTCTC-3'<br><br>Reverse: 5'-TTTGCTTACGGGATCCTCCTTTCCCCTCGCTCAGCA-3'
pUAST	*UAS-otopla*	Forward: 5'-CGCTCATATGGAATTCATGGGCGGCGGTGAAGTGAAG-3'<br><br>Reverse:<br>5' TAGAGGTACCCTCGAGTTACTCCAGACGTGCCTTGTAGGTG-3'
pUAST	*UAS-otoplaE638A*	Forward: 5'-CGCCCCGATCAGCGCATAC(T→G)CGATGATGAACGGA-3'<br><br>Reverse: 5'-TCCGTTCATCATCG(A→C)GTATGCGCTGATCGGGGCG-3'
pcDNA3	*pcDNA3-otopla*	Forward: 5'-TTGCGGCCGCGAATTCATGGGCGGCGGTGAAGTGAAG-3'<br><br>Reverse:<br>5'-TGGTGGCGATGGATCCCTCCAGACGTGCCTTGTAGGTG-3'
pCS2+MT	*pCS2+MT-otopla*	Forward: 5'-AGAGGACTTGAATTCAATGGGCGGCGGTGAAGTGAAG-3'<br><br>Reverse:<br>5'-GTTCTAGAGGCTCGAGTTACTCCAGACGTGCCTTGTAGGTG-3'
pGEX6-p-1	*pGEX6-p-1-otopla antigen*	Forward: 5'-GGGGCCCCTGGGATCCGCCCACTCGATTCGTCAGC-3'<br><br>Reverse:<br>5'-GATGCGGCCGCTCGAGTTACACATTGTCGCTCTTGTACACAT-3'
