# Supplementary material for: Molecular and cellular basis of acid taste sensation in Drosophila
Source: Nat Commun. 2021 Jun 17;12:3730. doi: 10.1038/s41467-021-23490-5 (PMC8211824; doi:10.1038/s41467-021-23490-5)
Supplement: Supplementary file 2 — Reporting Summary [file 41467_2021_23490_MOESM2_ESM.pdf]

nature research

Corresponding author(s): Yali Zhang

Last updated by author(s): April 12, 2021

Reporting Summary

Nature Research wishes to improve the reproducibility of the work that we publish. This form provides structure for consistency and transparency in reporting. For further information on Nature Research policies, see our Editorial Policies and the Editorial Policy Checklist.

Statistics

For all statistical analyses, confirm that the following items are present in the figure legend, table legend, main text, or Methods section.

n/a	Confirmed	
[ ]	[x]	The exact sample size (n) for each experimental group/condition, given as a discrete number and unit of measurement
[ ]	[x]	A statement on whether measurements were taken from distinct samples or whether the same sample was measured repeatedly
[ ]	[x]	The statistical test(s) used AND whether they are one- or two-sided<br>*Only common tests should be described solely by name; describe more complex techniques in the Methods section.*
[x]	[ ]	A description of all covariates tested
[x]	[ ]	A description of any assumptions or corrections, such as tests of normality and adjustment for multiple comparisons
[ ]	[x]	A full description of the statistical parameters including central tendency (e.g. means) or other basic estimates (e.g. regression coefficient) AND variation (e.g. standard deviation) or associated estimates of uncertainty (e.g. confidence intervals)
[ ]	[x]	For null hypothesis testing, the test statistic (e.g. F, t, r) with confidence intervals, effect sizes, degrees of freedom and P value noted<br>*Give P values as exact values whenever suitable.*
[x]	[ ]	For Bayesian analysis, information on the choice of priors and Markov chain Monte Carlo settings
[x]	[ ]	For hierarchical and complex designs, identification of the appropriate level for tests and full reporting of outcomes
[x]	[ ]	Estimates of effect sizes (e.g. Cohen's d, Pearson's r), indicating how they were calculated

Our web collection on statistics for biologists contains articles on many of the points above.

Software and code

Policy information about availability of computer code

Data collection	Patch clamp data were recorded using the commercial software Clampex 10.6. Tip recording data were collected using the commercial software Autospike32 v3.9. No codes were used.
Data analysis	Patch clamp data was analyzed using Clampfit 10.6. Tip recording data was analyzed using Autospike32 v3.9. No codes were used.

For manuscripts utilizing custom algorithms or software that are central to the research but not yet described in published literature, software must be made available to editors and reviewers. We strongly encourage code deposition in a community repository (e.g. GitHub). See the Nature Research guidelines for submitting code & software for further information.

Data

Policy information about availability of data

All manuscripts must include a data availability statement. This statement should provide the following information, where applicable:

- Accession codes, unique identifiers, or web links for publicly available datasets
- A list of figures that have associated raw data
- A description of any restrictions on data availability

All relevant data are available in the manuscript and supplementary information, or are available upon request from the corresponding author.

Field-specific reporting

Please select the one below that is the best fit for your research. If you are not sure, read the appropriate sections before making your selection.

- [x] Life sciences
- [ ] Behavioural & social sciences
- [ ] Ecological, evolutionary & environmental sciences

For a reference copy of the document with all sections, see nature.com/documents/nr-reporting-summary-flat.pdf

Life sciences study design

All studies must disclose on these points even when the disclosure is negative.

Sample size	The sample sizes were determined based on the LaMorte's power calculations. We chose a p value less than 0.05 and a power level of 80%. In this study, about 70 flies were used for each trial in the two-way feeding assays. At least 10 flies were used for each trial in the proboscis extension reflex (PER) assays, at least 8 flies for the taste electrophysiological assays, and at least 7 cells for the patch clamp assays. Moreover, these sample sizes have been successfully used in the statistics of our previous publications (Zhang et al., Science, 2013a; Zheng et al., Nature Neuroscience, 2013b; Zhang et al., Neuron, 2016).
Data exclusions	When we conducted in vivo taste electrophysiology, flies were tested with 50 mM sucrose solution after recording, to ensure the animals remained in good physical condition. If the fly did not fire action potentials to the sucrose stimulus, this indicated a potential physiological defect, and we did not use that animal for further statistical analyses. These exclusion standards have been established by our previous work (Zhang et al., Science, 2013a; Zhang et al., Nature Neuroscience, 2013b; Zhang et al., Neuron, 2016).
Replication	The number of replicates required was based on LaMorte's power calculations, with the power set at 0.8 and the standard significance value set at 0.05. For each assay, at least three replicates were performed. All attempts at replication were successful.
Randomization	For all the behavioral and physiological tests, flies of each genotype, such as wild-type, otopla mutant, and rescue flies, were collected randomly from the fly bottles where we breed the flies. In the patch clamp analysis, the HEK293 cells expressing the GFP reporters were chosen randomly with the epifluorescence microscope.
Blinding	To avoid bias in the feeding and physiological assays, flies or cells were grouped and encoded by one researcher and then used by a different researcher to record and collect data. The genotypes of the flies and cells were not decrypted until the analysis was complete.

Reporting for specific materials, systems and methods

We require information from authors about some types of materials, experimental systems and methods used in many studies. Here, indicate whether each material, system or method listed is relevant to your study. If you are not sure if a list item applies to your research, read the appropriate section before selecting a response.

Materials & experimental systems

n/a	Involved in the study	
[ ]	[x]	Antibodies
[ ]	[x]	Eukaryotic cell lines
[x]	[ ]	Palaeontology and archaeology
[ ]	[x]	Animals and other organisms
[x]	[ ]	Human research participants
[x]	[ ]	Clinical data
[x]	[ ]	Dual use research of concern

Methods

n/a	Involved in the study	
[x]	[ ]	ChIP-seq
[x]	[ ]	Flow cytometry
[x]	[ ]	MRI-based neuroimaging

Antibodies

Antibodies used	Rabbit anti-GFP polyclonal antibody (1:200; Thermo Fisher, Cat. No. A-11122, RRID_AB_221569), mouse anti-nc82 monoclonal antibody (1:50; Developmental Studies Hybridoma Bank (DSHB), Cat. No. nc82, RRID_AB_528108), mouse anti-c-Myc monoclonal antibody (clone 9E10; 1:100; Thermo Fisher, Cat. No. MA1-980, RRID_AB_558470), rabbit anti-OtopLa polyclonal antibody (1:200; generated in this study), mouse anti-mCherry monoclonal antibody (1:100; DSHB, Cat. No. DSHB-mCherry-3A11, RRID_AB_2617430), goat anti-rabbit Alexa Fluor 488 (1:200; Jackson ImmunoResearch, Cat. No. 111-585-003, RRID_AB_2338059), goat anti-mouse Alexa Fluor 594 (1:200; Jackson ImmunoResearch, Cat. No. 115-545-003, RRID_AB_2338840), and donkey anti-mouse Alexa Fluor 594 (1:200; Jackson ImmunoResearch, Cat. No. 715-585-150, RRID_AB_2340854).

Validation	We have successfully performed immunocytochemical analyses of the fly proboscis and brains in our previous work (Zhang et al., Science, 2013a; Zhang et al., Nature Neuroscience, 2013b; Zhang et al., Neuron, 2016), using the following primary and secondary antibodies: rabbit anti-GFP polyclonal antibody (1:200; Thermo Fisher, Cat. No. A-11122, RRID_AB_221569), mouse anti-nc82 monoclonal antibody (1:50; Developmental Studies Hybridoma Bank (DSHB), Cat. No. nc82 RRID_AB_528108), mouse anti-mCherry monoclonal antibody (1:100; DSHB, Cat.No. DSHB-mCherry-3A11, RRID_AB_2617430), goat anti-rabbit Alexa Fluor 488 (1:200; Jackson ImmunoResearch, Cat. No. 111-585-003, RRID_AB_2338059), goat anti-mouse Alexa Fluor 594 (1:200; Jackson ImmunoResearch, Cat. No. 115-545-003, RRID_AB_2338840), and donkey anti-mouse Alexa Fluor 594 (1:200; Jackson ImmunoResearch, Cat. No. 715-585-150, RRID_AB_2340854).<br><br>In this study, the mouse anti-c-Myc monoclonal antibody (clone 9E1; 1:100; Thermo Fisher, Cat. No. MA1-980, RRID_AB_558470) was validated by our immunocytochemical assays of the HEK293 cells expressing the OtopLa fused with a Myc tag. The rabbit anti-OtopLa polyclonal antibody (1:200; generated in this study) was validated by our immunocytochemical analysis of the wild-type and otopla1 mutant flies.

Eukaryotic cell lines

Policy information about cell lines

Cell line source(s)	HEK293 cells (Cat. No. ATCC® CRL-2828™, RRID_CVCL_0045) were obtained from the American Type Culture Collection (ATCC).
Authentication	The identify of this cell line was confirmed by morphological and karyotyping tests.
Mycoplasma contamination	No mycoplasma contaminations were found in cultured HEK293 cells.
Commonly misidentified lines (See ICLAC register)	No commonly misidentified cell lines were used in this study.

Animals and other organisms

Policy information about studies involving animals; ARRIVE guidelines recommended for reporting animal research

Laboratory animals	All of the following are fly lines: otopla1 (generated in this study), wild-type flies (w1118), UAS-otopla (generated in this study), UAS-otoplaE628A (generated in this study), UAS-otopla RNAi (RRID_VDRC_104973), UAS-otoplb RNAi (RRID_VDRC_101936), UAS-otoplc RNAi (RRID_VDRC_108591), Otopla-Gal4 (generated in this study), Gr5a-LexA-VP16, Gr5a-Gal4 (RRID_BDSC_57592), Gr66a-LexA-VP16, Gr66a-Gal4 (RRID_BDSC_57670), poxn-Gal4 (RRID_BDSC_66685), Ir76b-QF, UAS-mCD8::GFP (RRID_BDSC_5137), QUAS-mtdTomato (RRID_BDSC_30005), and LexOP-mCherry-HA (RRID_BDSC_52271). Both male and female flies of two-four-day old were used in this study.
Wild animals	This study did not use wild animals.
Field-collected samples	This study did not use field-collected samples.
Ethics oversight	No ethical approval was required for this study, as research using drosophila does not require IACUC oversight.

Note that full information on the approval of the study protocol must also be provided in the manuscript.
